# Structural and functional brain alterations in depression with Alzheimer’s disease and mild cognitive impairment: a multimodal coordinate-based meta-analysis

**DOI:** 10.3389/fnagi.2026.1784966

**Published:** 2026-03-26

**Authors:** Yang Du, Song Li, Junlin Diao, Biao Du, Guoqing Jiang

**Affiliations:** 1 Center for Sleep Medicine, Chongqing Mental Health Center, Chongqing, China; 2 Department of Pharmacy, Chongqing Mental Health Center, Chongqing, China; 3 Department of Pharmacy, Chongqing University Three Gorges Hospital, Chongqing, China

**Keywords:** activation likelihood estimate, Alzheimer’s disease, coordinate-based meta-analyses, depression, mild cognitive impairment

## Abstract

**Background:**

Depression is a common neuropsychiatric symptom in Alzheimer’s Disease (AD) and mild cognitive impairment (MCI). Inconsistent results for comparison between AD with depression (ADD), MCI with depression (MCID), and those without depression were obtained from previous neuroimaging studies. Therefore, a multimodal coordinate-based meta-analysis was conducted in this study.

**Methods:**

We searched PubMed, Embase, PsycINFO and the Cochrane Library for neuroimaging studies on ADD and MCID patients from December 1995 to December 2025. Activation likelihood estimate (ALE) coordinate-based meta-analyses were performed to explore brain regions showing abnormalities in ADD and MCID compared to AD without depression (ADND) and MCI without depression (MCIND). Moreover, we performed modality and subgroup analysis of the difference in structural and neural activity.

**Results:**

A total of 30 studies, comprising 1973 participants (520 ADD patients, 297 MCID patients, 748 ADND patients, and 408 MCIND patients), were included in this meta-analysis. ADD and MCID showed significantly decreased gray matter volume (GMV) in the right hippocampus and neural activity in the right superior frontal gyrus (SFG) and left inferior temporal gyrus (ITG) compared to ADND and MCID. Subgroup analysis revealed decreased neural activity in the left SFG in the ADD group compared to the ADND group, and decreased neural activity in the right SFG and left ITG in the MCID group compared to the MCIND group.

**Conclusion:**

Our findings that decreased GMV of the hippocampus and abnormal neural activities of SFG and ITG in ADD and MCID expand novel insights into the neural mechanisms of depression in AD and MCI, representing potential neural correlates for depression in ADD and MCID.

## Introduction

Alzheimer’s disease (AD), the most common cause of dementia, is characterized by progressive memory loss and deficits in executive functions, language, decision-making, and visuospatial abilities (Lane et al., 2018). Mild cognitive impairment (MCI) is the intermediate stage between normal aging and dementia, especially AD (Giau et al., 2019). Neuropsychiatric symptoms (NPS) are a common clinical manifestation of AD and MCI, which can cause significant distress to patients and their caregivers (Lanctot et al., 2017). Of note, depression is one of the common NPS in AD and MCI. Several studies showed that the prevalence of depression in patients with AD and MCI ranges from 37 to 46% (Leung et al., 2021; Zhao et al., 2016). Most importantly, there is growing evidence that the comorbidity with depression in AD and MCI is more likely to accelerate cognitive decline and lead to poorer functional outcomes (Gallagher et al., 2018). Despite the high prevalence and clinical relevance, the underlying mechanism of AD patients with depression (ADD) and MCI patients with depression (MCID) is not well understood. Therefore, understanding the underlying neurobiological mechanisms in ADD and MCID patients is essential for developing effective interventions.

To elucidate the neurobiological basis of ADD and MCID, numerous neuroimaging studies have employed voxel-based morphometry (VBM), resting-state functional magnetic resonance imaging (rs-fMRI), single-positron emission computed tomography (SPECT), and positron emission tomography (PET) techniques to investigate abnormalities in brain structure and function (Radmard et al., 2025). Evidence from prior structural and functional neuroimaging studies demonstrated that AD is closely associated with severe atrophy and dysfunction in the hippocampus and posterior cingulate cortex (Talwar et al., 2021). MCI is associated with dysfunction in the default mode network (Yan et al., 2019), while depression is linked to dysfunction in the superior frontal gyrus and ventromedial frontal cortex (Boccia et al., 2015). Structural neuroimaging revealed a specific pattern of brain atrophy, whereas functional neuroimaging demonstrated alterations in neural activity, providing useful evidence for differential diagnosis. Several VBM studies have found that ADD patients may be associated with greater regional atrophy in the frontal lobe, compared to ADND patients (Sinclair et al., 2023). However, another study found no difference between ADD and ADND patients (Langella et al., 2024). In light of these inconsistent results, coordinate-based meta-analysis plays a critical role in neuroimaging research.

Rs-fMRI has long been used to assess brain activity in neuropsychiatric disorders due to its non-invasive and task-free nature (Takamura and Hanakawa, 2017). Previous rs-fMRI studies have found that ADD patients, compared to ADND patients, demonstrate extensive abnormal dysfunction with a range of brain regions involved in the superior frontal gyrus, medial prefrontal cortex, and supplementary motor area (Zhang et al., 2017; Liu et al., 2017a). Moreover, evidence suggests that abnormalities in affective network dysfunction, including amygdala-medial prefrontal cortex and amygdala-sensorimotor networks, are more pronounced in MCID patients than in MCIND patients, which may be involved in the neural mechanism underlying depression in patients with MCID (Guan C. et al., 2022; Yang et al., 2021). However, previous neuroimaging findings demonstrating various abnormalities in resting-state activity in ADD and MCID patients have yielded inconsistent results.

The development of perfusion imaging techniques such as SPECT and PET offers considerable scope for investigating brain metabolic changes in AD and MCI (Burgio et al., 2024). There is evidence that hypoperfusion in the prefrontal area and hyperperfusion in the supplementary motor area may be associated with the expression of depressive symptoms in AD (Li et al., 2021). In addition, it is shown that MCID patients presenting abnormalities in frontal and parietal metabolic patterns are more likely to progress to dementia (Mauri et al., 2022). Generally, although many studies have attempted to identify differences in cerebral metabolism between AD and MCI with depression and those without depression, the results of these studies were inconsistent. The discrepant findings of prior studies may be attributed to the small sample sizes and clinical heterogeneity, which reminds us of the necessity of performing quantitative meta-analysis to further identify specific neural mechanisms of depression in ADD and MCID.

A meta-analysis combines the results of independently conducted studies to increase power and yield results that are more reproducible than those of the original studies (Higgins et al., 2003). Coordinate-based meta-analysis is the primary approach for analyzing neuroimaging research (Samartsidis et al., 2017). Activation likelihood estimation (ALE), a commonly used coordinate-based meta-analysis method, typically calculates the union of reported results across locations (Eickhoff et al., 2012). Several previous meta-analyses focused on AD and MCI patients without considering depression, which have found impaired functional activity in frontoparietal and default networks. To our knowledge, no meta-analysis that combined different modalities has explored the difference in structural and neural activity in ADD and MCID patients and ADND and MCID patients. Hence, we aimed to perform ALE meta-analyses to comprehensively examine the abnormalities in structural and neural activity in ADD and MCID compared to ADND and MCI. In addition, we performed analyses of differences in structural and neural activity across modalities and subgroups.

## Methods

### Search strategies

The present meta-analysis has been registered in PROSPERO (ID: CRD42025 1,279,608). We conducted the systematic reviews and meta-analyses following the Preferred Reporting Items for Systematic Reviews and Meta-Analyses (PRISMA) statement (Tricco et al., 2018). We systematically searched PubMed, Web of Science, MEDLINE, EMBASE, PsycINFO, and the Cochrane Library for publications from December 1995 to December 2025. We included neuroimaging studies in ADD or MCID patients reporting altered brain activity compared with ADND or MCIND patients. The search strategies are different combinations of the following terms, including “Alzheimer’s disease, AD, mild cognitive impairment, MCI, depression, structural MRI, VBM, functional MRI, rs-fMRI, neuroimaging, functional imaging, functional connectivity density (FCD), regional homogeneity (ReHo), the amplitude of low-frequency fluctuations (ALFF) and fractional ALFF (fALFF), Positron Emission Computed Tomography (PET), Single-Photon Emission Computed Tomography (SPECT)”. Further exploration of the relevant reviews and meta-analyses was conducted to identify original studies that may have been missed in the above search.

### Selection criteria

From these research articles, we included whole-brain comparative studies meeting the following criteria: (1) the full text was published in English; (2) structural and functional neuroimaging alternations related to the comparison between ADD and ADND groups or MCID and MCIND groups; (3) scans without a predefined stimulus or task; (4) the Montreal Neurological Institute (MNI) or Talairach coordinates reported differences in voxel signal intensity. Structural MRI included the whole-brain analysis using voxel-based morphometry (VBM), and functional MRI included whole-brain functional connectivity, PET, and SPECT.

Studies were excluded if (1) they were seed-based or region-of-interest (ROI) based correlational analysis; (2) the peak coordinates of the effect could not be obtained after contacting the author via e-mail; (3) reviews, letters, conference abstracts, and case reports were omitted; (4) articles did not report specific changes at a regional or voxel level.

### Quality assessment and data extraction

We used a 12-point checklist (Zhao et al., 2022) to assess the quality of individual studies, focusing on clinical and demographic aspects of the study sample and the imaging methodology (Supplementary Tables S1, S2). Two authors (YD, SL) independently reviewed the retrieved literature and determined whether each study should be included. The researchers agreed to interpret each item of the 12-point checklist based on previous meta-analyses and to assess the studies using the same methodology. Any conflicts that arose during the review were discussed and resolved by YD and SL.

YD and GQJ extracted data from each eligible article. These authors independently extracted information from all included studies, including the first author, year of publication, cohort size, age, sex, education level, age at onset, illness duration, Mini-Mental State Examination (MMSE) scores, 3D coordinates, the number of foci, and the contrasts of the included studies. If a study contained multiple independent patient samples, we extracted the appropriate coordinates into two separate experiments.

### Coordinate-based meta-analysis

#### Main meta-analysis

We used GingerALE version 3.0.2^1^ to perform coordinate-based ALE analysis. The ALE approach, a widely used technique for synthesizing neuroimaging data, pools coordinates in a three-dimensional stereotactic space derived from neuroimaging experiments and spatially normalizes them to a single template (Eickhoff et al., 2012). First, we import Talairach space coordinates for ADD and MCID patients, compared with ADND and MCIND patients, into the software. If the coordinates are reported in MNI space, we use the ‘Convert Foci’ tool based on the ‘icbm2tal’ algorithm to convert them to Talairach space. Second, ALE calculations create a three-dimensional image for each foci group, the foci, and a Gaussian blur with a full-width-half-maximum (FWHM) empirically derived from subject size, called Modelled Activation (MA) maps (Eickhoff et al., 2009). Next, the union of the MA maps for each experiment is created to form an ALE image that contains the combined probability distribution of activation at each voxel. Evidence indicates that the cluster-level family-wise error (FWE) correction is the most appropriate method for statistical inference regarding multiple-comparison errors in ALE meta-analyses (Frahm et al., 2022; Eickhoff et al., 2016). We followed the ALE best practices (Tahmasian et al., 2019; Muller et al., 2018) and corrected the results using a cluster-forming threshold of *p* < 0.001 and a cluster-level FWE threshold of *p* < 0.05, based on 1,000 random permutations of the input data features. Finally, we used Mango software version 3.1.2^2^ to visualize the results registered on a standardized Talairach anatomical template.

#### Jackknife sensitivity analysis

Substantial variability indicates that the results are driven by specific neglected studies, compromising the reliability of the findings and rendering them vulnerable to false discoveries. Therefore, we conducted jackknife sensitivity analyses by repeatedly performing the same analyses while excluding one dataset each time to test the consistency of results across different studies (Pan et al., 2017).

#### Subgroup meta-analysis

A recent study has revealed that brain structure and function exhibit a progressive, gradient deterioration pattern, transitioning from normal aging stages through MCI to AD (Zhao et al., 2024), suggesting that each disorder exhibits unique dysfunctional neurophysiological characteristics. To identify the potential effects of varying degrees of cognitive impairment in AD and MCI on structure and neural activity, we conducted subgroup meta-analyses of studies comparing patients with ADD and ADND, and studies comparing patients with MCID and MCIND, respectively. To perform complementary analyses on more homogeneous subsections of the data (Salimi-Khorshidi et al., 2009), we split experiments showing decreases (ADD < ADND), experiments showing increases (ADD > ADND), experiments showing decreases (MCID < MCIND), and experiments showing increases (MCID > MCIND). In the exploratory meta-analyses, to perform complementary analyses on more homogeneous subsections of the data, we split the experiments by imaging modalities, including structural and functional imaging. The meta-analysis conducted additional subgroup analyses to identify modality-specific and subgroup-specific convergence in more homogeneous data. Therefore, we performed modality-specific and subgroup-specific analyses of differences in structure and neural activity.

## Results

**Figure 1:**
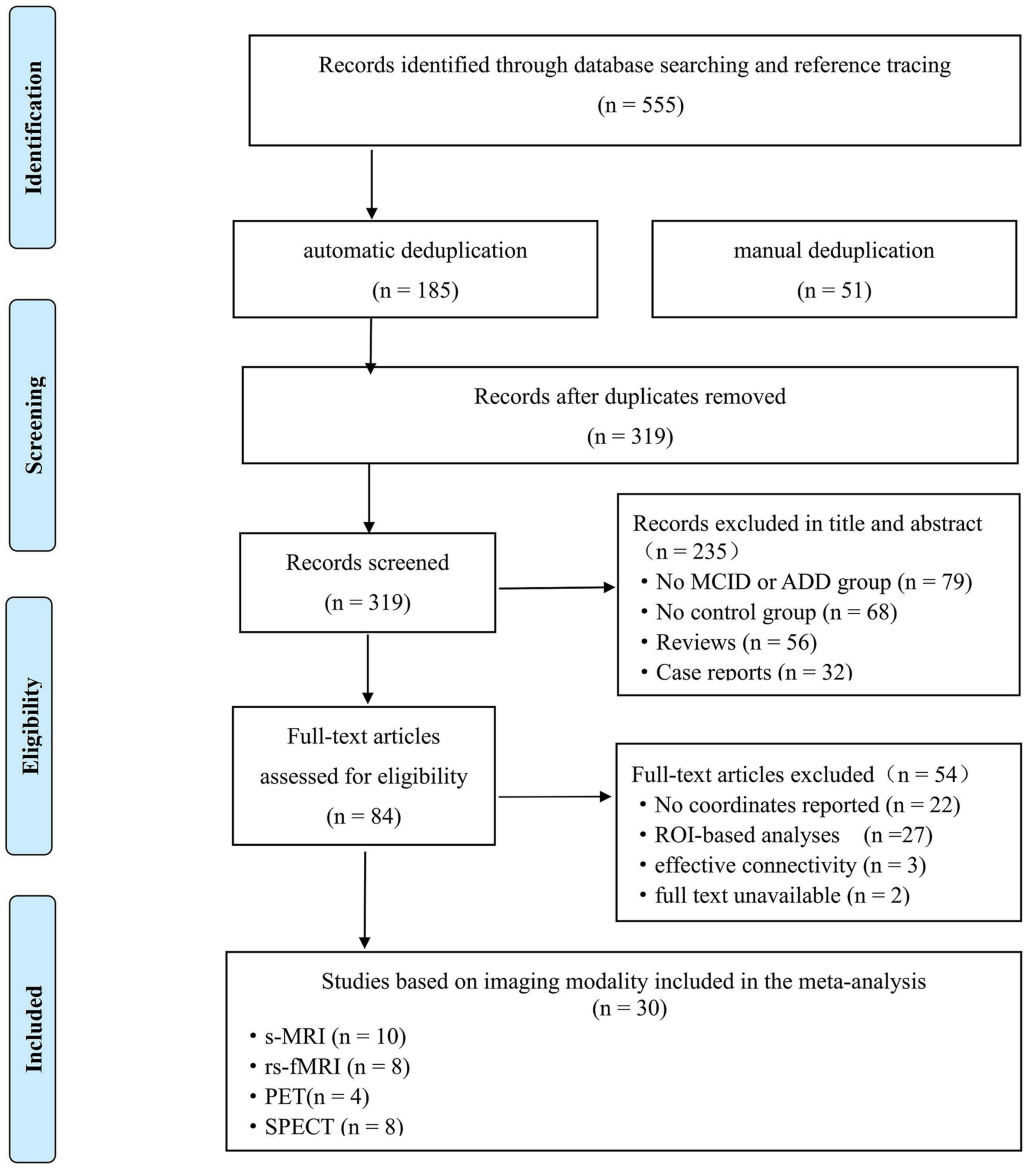
Flow diagram showing the results of the systematic search for the selected studies in the activation likelihood estimate coordinate-based meta-analysis.

Figure 1 shows the selection process based on the PRISMA statement. The initial search identified 555 studies. We performed a rigorous duplicate removal process to ensure the integrity of our meta-analysis dataset. Initially, potential duplicates were identified using EndNote’s automatic duplicate detection function, which screened citation fields including author, year, and titles. This automated procedure flagged and removed 185 duplicate records. Subsequently, a manual review was conducted to verify and eliminate any additional duplicates that may have been missed by the automated process. During this step, 51 references were carefully examined, ensuring that no duplicate entries remained. In total, 236 duplicate records were excluded from the 555 initially retrieved articles. Details of the procedure are provided in Supplementary material 2. After removing the 236 duplicates, 319 studies remained. Titles and abstracts were screened to rule out irrelevant studies, and the full texts of 84 available studies were reviewed. After applying the inclusion and exclusion criteria, 30 studies met the inclusion criteria at the full-text assessment and were considered eligible, two of which conducted separate analyses within the same study. Notably, we did not identify any suitable original research after further examination of relevant reviews and meta-analyses. Moreover, our search strategy is shown in Supplementary Table S3. The total sample covering 1973 participants comprised 520 patients with ADD, 748 patients with ADND, 297 patients with MCID, and 408 patients with MCIND. The 30 studies included 10 VBM studies and 20 functional imaging studies, including 8 whole-brain fMRI studies, 8 SPECT studies, and 4 PET studies, which we included in the ALE meta-analysis. Specifically, seventeen studies compared ADD patients with ADND patients, including six VBM studies (Son et al., 2013; Lebedeva et al., 2014; Hu et al., 2015; Karavasilis et al., 2017; Mohamed Nour et al., 2021; Querry et al., 2024), four fMRI studies (Guo et al., 2015; Liu et al., 2017b; Guo et al., 2017; Mu et al., 2020), seven SPECT studies (Liao et al., 2003; Levy-Cooperman et al., 2008; Kang et al., 2012; Honda et al., 2014; Terada et al., 2014; Oshima et al., 2014; Hayashi et al., 2016), and two PET studies (Holthoff et al., 2005; Lee et al., 2006), while nine studies including nine experiments compared MCID patients with MCIND patients, including four VBM studies (Xie et al., 2012; Lyu et al., 2019; Du et al., 2022; Chen et al., 2025), four fMRI studies (Li et al., 2017; Liu et al., 2018; Yu et al., 2019; Liu et al., 2019), one SPECT study (Hirao et al., 2022) and two PET study (Lee et al., 2010; Brendel et al., 2015). Demographic, clinical, and methodological details extracted from the studies are reported in Table 1. Moreover, medication use of each study is summarized in Supplementary Table S4.

**Table 1:** List of included studies on structure and neural activity in ADD and MCID compared to ADND and MCIND.

Study	Method	Number	Age	Sex (M/F)	Education	MMSE	Depression measure	Contrasts	Foci
Structural imaging									
Son et al. (2013)	VBM	32 ADND	76.1 (4.9)	12/20	5.0 (3.8)	18.0 (3.4)	GDS	ADND > ADD	1
		17 ADD	77.0 (8.3)	7/10	5.4 (3.4)	17.7 (3.5)	/	ADD > ADND	0
Lebedeva et al. (2014)	VBM	89 ADND	75.0 (5.1)	49/40	12.0 (4.1)	24.0 (2.7)	GDS/CSDD	ADND > ADD	6
		100 ADD	76.0 (6.3)	57/43	12.4 (4.4)	22.7 (3.2)	/	ADD > ADND	0
Hu et al. (2015)	VBM	45 ADND	75.1 (6.7)	24/21	15.7 (3.0)	23.0 (2.6)	NPI-Q	ADND > ADD	4
		40 ADD	76.5 (7.1)	22/18	15.4 (3.4)	22.5 (2.8)	/	ADD > ADND	0
Karavasilis et al. (2017)	VBM	120 ADND	74.7 (7.3)	40/80	10.9 (4.2)	21.0 (2.8)	GDS	ADND > ADD	12
		53 ADD	72.7 (9.0)	11/42	9.3 (4.7)	20.7 (3.9)	/	ADD > ADND	0
Mohamed Nour et al. (2021)	VBM	35 ADND	76.9 (6.5)	20/15	15.3 (3.2)	23.6 (2.2)	NPI-Q	ADND > ADD	6
		19 ADD	73.3 (8.2)	6/13	14.8 (3.5)	23.5 (1.8)	/	ADD > ADND	0
Querry et al. (2024)	VBM	20 ADND	69.9 (9.5)	9/11	11.6 (4.5)	22.7 (2.8)	MINI	ADND > ADD	1
		17 ADD	71.4 (9.9)	5/12	12.7 (3.7)	23.4 (2.7)	/	ADD > ADND	0
Xie et al. (2012)	VBM	17 MCIND	75.1 (6.6)	11/6	13.5 (2.1)	27.2 (1.8)	GDS	MCI > MCID	9
		12 MCID	74.8 (9.8)	7/5	14.2 (2.9)	26.9 (1.9)	DSM IV	MCID > MCI	0
Lyu et al. (2019)	VBM	17 MCIND	61.7 (6.9)	9/8	8.7 (3.8)	18.8 (1.9)	HAMD	MCI > MCID	3
		18 MCID	65.8 (7.2)	8/10	7.6 (3.7)	20.2 (2.3)	/	MCID > MCI	0
Du et al. (2022)	VBM	70 MCIND	75.9 (6.9)	38/32	16.4 (2.5)	25.8 (2.8)	GDS	MCI > MCID	2
		70 MCID	75.8 (7.3)	40/30	16.3 (2.8)	27.0 (1.7)	/	MCID > MCI	0
Chen et al. (2025)	VBM	15 MCIND	61.4 (6.9)	7/8	7.8 (3.9)	26.3 (2.5)	HAMD	MCI > MCID	2
		16 MCID	64.3 (6.0)	10/6	9.2 (3.8)	24.7 (1.4)	/	MCID > MCI	0
Functional imaging									
Liao et al. (2003)	SPECT	35 ADND	75.2 (8.2)	7/26	2.8 (5.8)	12.8 (5.7)	HDRS	ADND > ADD	5
		8 ADD	72.9 (4.7)	2/6	5.7 (5.5)	13.3 (4.0)	DSM-III-R	ADD > ADND	0
Holthoff et al. (2005)	PET	10 ADND	63.2 (10.1)	4/6	10.5 (1.4)	21.8 (3.3)	NPI/HAMD	ADND > ADD	2
		10 ADD	69.5 (10.0)	4/6	11.4 (3.3)	22.5 (3.3)	/	ADD > ADND	0
Lee et al. (2006)	PET	12 ADND	70.6 (5.6)	0/12	7.5 (6.1)	21.9 (2.9)	CERAD	ADND > ADD	1
		12 ADD	71.3 (5.8)	0/12	6.1 (5.3)	21.2 (1.5)	PDD-AD	ADD > ADND	0
Levy-Cooperman et al. (2008)	SPECT	29 ADND	75.5 (4.8)	16/13	13.8 (4.0)	24.0 (3.6)	CSDD	ADND > ADD	2
		27 ADD	78.3 (7.2)	9/18	13.4 (3.0)	23.2 (4.4)	/	ADD > ADND	0
Kang et al. (2012)	SPECT	9 ADND	75.2 (4.4)	0/9	6.2 (6.5)	16.8 (4.6)	NPI	ADND > ADD	3
		9 ADD	74.5 (5.7)	0/9	7.3 (6.2)	16.3 (4.7)	/	ADD > ADND	0
Oshima et al. (2014)	SPECT	34 ADND	76.0 (6.0)	10/24	11.2 (2.1)	20.7 (4.8)	NPI/GDS	ADND > ADD	1
		23 ADD	76.0 (6.2)	8/15	10.7 (3.0)	21.0 (3.9)	/	ADD > ADND	0
Terada et al. (2014)	SPECT	51 ADND	76.2 (7.6)	23/28	11.0 (2.5)	21.4 (4.2)	NPI	ADND > ADD	2
		28 ADD	76.2 (7.6)	11/17	11.0 (2.5)	21.4 (4.2)	/	ADD > ADND	0
Honda et al. (2014)	SPECT	46 ADND	76.9 (7.2)	18/28	11.0 (2.4)	21.3 (4.3)	NPI/GDS	ADND > ADD	1
		30 ADD	75.2 (8.3)	14/16	10.9 (2.4)	21.8 (4.0)		ADD > ADND	0
Guo et al. (2015)	ReHo	17 ADND	75.8 (3.8)	8/9	8.5 (1.9)	21.0 (2.1)	NPI/HAMD	ADND > ADD	4
		15 ADD	73.5 (4.7)	6/9	8.6 (1.8)	19.6 (2.2)	/	ADD > ADND	0
Hayashi et al. (2016)	SPECT	70 ADND	76.5 (8.2)	29/41	10.9 (2.7)	21.3 (2.4)	NPI/GDS	ADND > ADD	3
		46 ADD	75.6 (7.4)	17/29	10.1 (1.9)	21.6 (1.8)	/	ADD > ADND	0
Guo et al. (2017)	fALFF	21 ADND	73.9 (5.2)	11/10	9.1 (2.4)	20.4 (1.7)	NPI/HAMD	ADND > ADD	1
		22 ADD	71.9 (4.5)	11/11	9.7 (2.2)	20.9 (2.3)	/	ADD > ADND	3
Liu et al. (2017a,b)	ALFF	21 ADND	73.9 (5.2)	11/10	9.1 (2.4)	20.4 (1.7)	NPI/HAMD	ADND > ADD	1
		22 ADD	71.9 (4.5)	11/11	9.7 (2.2)	20.9 (2.3)	/	ADD > ADND	2
Mu et al. (2020)	ALFF	52 ADND	74.7 (3.6)	29/23	8.1 (2.1)	15.8 (2.2)	HAMD	ADND > ADD	4
		22 ADD	72.4 (4.1)	9/13	7.6 (2.4)	14.6 (3.1)	/	ADD > ADND	0
Lee et al. (2010)	PET	18 MCIND	69.4 (10.1)	4/14	8.6 (5.0)	26.9 (1.9)	HDRS	MCI > MCID	1
		18 MCID	69.4 (9.5)	6/12	9.7 (4.0)	24.2 (2.5)	/	MCID > MCI	0
Brendel et al. (2015)	PET	141 MCIND	73.4 (6.8)	75/66	16.1 (2.8)	27.8 (1.9)	NPI-Q	MCI > MCID	5
		65 MCID	73.7 (7.3)	34/31	16.2 (2.6)	27.6 (1.7)	/	MCID > MCI	1
Li et al. (2017)	ALFF	27 MCIND	67.4 (8.5)	13/14	10.3 (4.9)	23.5 (3.3)	HDRS	MCI > MCID	3
		19 MCID	65.8 (10.3)	9/10	9.2 (5.7)	24.6 (4.0)	DSM-IV	MCID > MCI	2
Liu et al. (2018)	ALFF	18 MCIND	72.1 (9.7)	7/11	8.5 (1.8)	26.6 (1.0)	NPI/HAMD	MCI > MCID	1
	FCD	16 MCID	69.6 (6.2)	6/10	8.3 (2.1)	26.6 (1.1)	DSM-IV	MCID > MCI	2
Yu et al. (2019)	ALFF	49 MCIND	65.9 (9.8)	19/30	9.8 (3.6)	25.4 (3.5)	GDS	MCI > MCID	8
	ReHo	27 MCID	63.4 (10.6)	11/16	9.4 (3.1)	25.0 (4.1)	DSM-V	MCID > MCI	8
Liu et al. (2019)	ReHo	18 MCIND	72.1 (9.7)	7/11	8.5 (1.8)	26.6 (1.0)	NPI/HAMD	MCI > MCID	2
		16 MCID	69.6 (6.2)	6/10	8.3 (2.1)	26.6 (1.1)	/	MCID > MCI	2
Hirao et al. (2022)	SPECT	18 MCIND	77.4 (5.6)	6/12	13.4 (2.3)	26.3 (1.6)	GDS	MCI > MCID	3
		20 MCID	76.5 (6.2)	7/13	14.4 (1.6)	26.7 (0.9)	/	MCID > MCI	1

MMSE, Mini-mental State Examination; SPECT, single-positron emission computed tomography; PET, positron emission tomography; ReHo, regional homogeneity; ALFF, amplitude of low-frequency fluctuations; fALFF, fractional amplitude of low-frequency fluctuations; FCD, functional connectivity density; ADD, AD with depression; ADND, AD without depression; MCID, MCI with depression; MCIND, MCI without depression; CSDD, Cornell Scale for Depression in Dementia; GDS, Geriatric Depression Scale; NPI-Q, Neuropsychiatric Inventory Questionnaire; NPI, Neuropsychiatric Inventory; MINI, The Mini International Neuropsychiatric Interview; HAMD, Hamilton Depression Rating Scale; HDRS, Hamilton Depression Rating Scale; CERAD, Consortium to Establish a Registry for Alzheimer disease; DSM, Diagnostic and Statistical Manual of Mental Disorders; PDD-AD, provisional diagnostic criteria for depression of AD.

**Figure 2:**
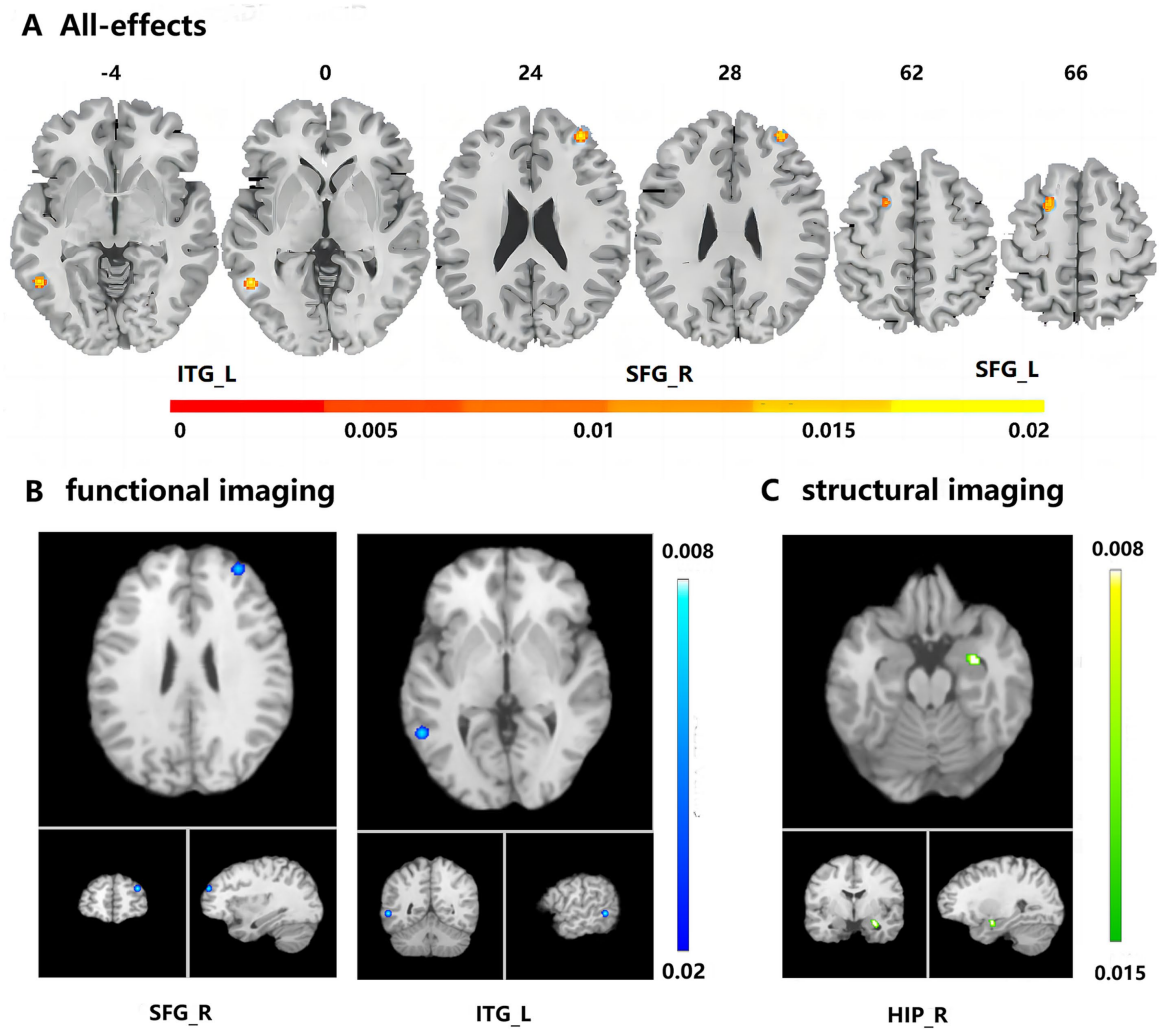
Abnormal regions identified in the activation likelihood estimate coordinate-based meta-analysis of neuroimaging studies in ADD and MCID patients compared to ADND and MCIND patients. **(A)** Multimodal meta-analysis results; **(B)** functional imaging results; **(C)** structural imaging results. ITG_L, left inferior temporal gyrus; SFG_R, right superior frontal gyrus; SFG_L, left superior frontal gyrus; HIP_R, right hippocampus.

The ALE analysis identified that ADD and MCID patients showed structural and functional abnormality in the left inferior temporal gyrus (ITG) (*p* = 7.28E-7, *Z* = 4.82) and right superior frontal gyrus (SFG) (*p* = 6.86E-7, *Z* = 4.83) and left SFG (*p* = 8.68E-6, *Z* = 4.30) and right hippocampus (*p* = 7.40E-6, *Z* = 4.07) compared with ADND and MCIND patients (Figure 2A). In the analysis for each modality, decreased neural activity resulted in significant cluster in the right SFG (*p* = 1.12E-8, *Z* = 5.59) and left ITG (*p* = 1.20E-8, *Z* = 5.57) in functional imaging analysis (Figure 2B), decreased GMV resulted in significant cluster in the right hippocampus (*p* = 7.41E-06, *Z* = 4.33) in structural imaging analysis (Figure 2C).

**Figure 3:**
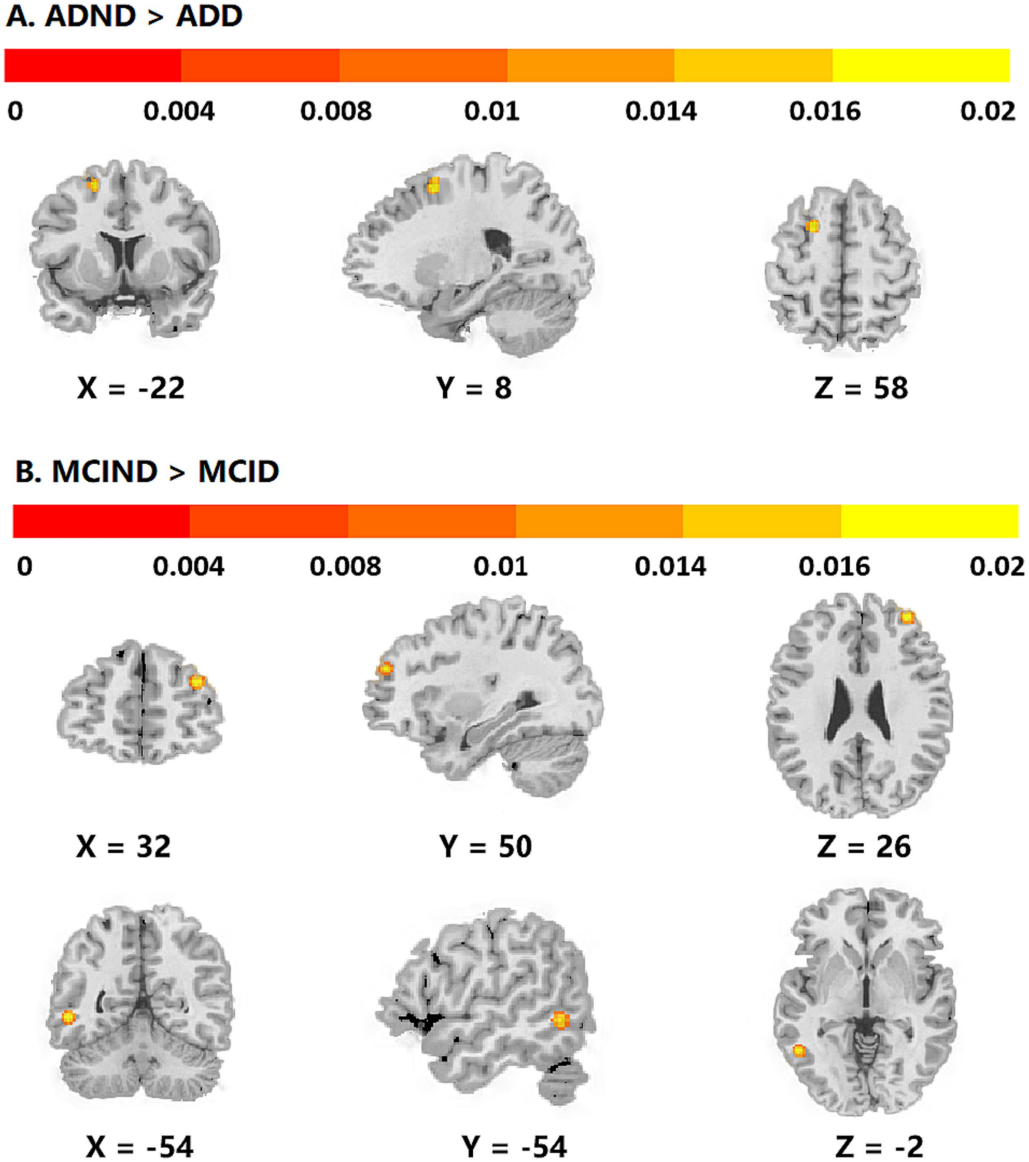
Subgroup analysis revealing abnormal regions identified in activation likelihood estimation based meta-analysis in ADD patients compared to ADND patients and MCID patients compared to MCIND patients. **(A)** Differences in brain structure and neural activation between ADND and ADD; **(B)** Differences in brain structure and neural activation between MCIND and MCID.

Subgroup analyses found that ADD patients showed structural and functional abnormality compared with ADND in the left SFG (*p* = 8.68E-6, *Z* = 4.30) (Figure 3A). In contrast, MCID patients showed structural and functional abnormalities compared with MCIND in the right SFG (*p* = 1.84E-9, *Z* = 5.90) and left ITG (*p* = 2.01E-9, *Z* = 5.88) (Figure 3B). ALE analysis identified significant clusters, and detailed information on coordinates and brain regions is shown in Table 2. Jackknife analysis results indicated that the VBM and fMRI meta-analyses showed high replicability and reliability (Supplementary Tables S5–S9).

**Table 2:** Results of ALE coordinate-based meta-analysis in structural and neural activity alterations in ADD and MCID compared with ADND and MCIND.

Analysis	Cluster	side	Brain region	BA	Coordinates (Talairach)			Cluster size (mm^3^)	ALE value	*Z* value
					X	Y	Z			
All-effects	1	Right	SFG	9	32	50	26	376	0.0203	4.83
	2	Left	SFG	6	−22	8	58	272	0.0170	4.30
	3	Left	ITG	37	−54	−54	−2	376	0.0202	4.82
	4	Right	HIP		30	−8	−20	264	0.0138	3.79
Subgroup										
ADND > ADD	1	Left	SFG	6	−22	8	58	536	0.0148	4.41
MCIND>MCID	1	Right	SFG	9	32	50	26	544	0.0203	5.77
	2	Left	ITG	37	−54	−54	−2	584	0.0202	5.76
ADND > ADD	No sig.									
MCIND>MCID	No sig.									
Modality										
Functional imaging	1	Right	SFG	9	32	50	26	520	0.0203	5.59
	2	Left	ITG	37	−54	−54	−2	552	0.0202	5.58
Structural imaging	1	Right	HIP		30	−8	−20	312	0.0116	4.07

ALE, activation likelihood estimation; BA, Brodmann area; SFG, superior frontal gyrus; ITG, inferior temporal gyrus; HIP, hippocampus.

## Discussion

To our knowledge, our study is the first to perform a comprehensive assessment of multimodal neuroimaging data to investigate the structural and functional abnormalities in ADD and MCID patients compared to ADND and MCIND patients. The ALE meta-analysis showed structural and functional alterations in the bilateral SFG, the right HIP, and the left ITG in ADD and MCID patients compared with ADND and MCIND patients. Subgroup analyses indicated that neural activity in the left SFG was decreased in patients with ADD compared to those with ADND, while neural activity in the right SFG and the left ITG were decreased in MCID patients compared to MCIND patients. We integrated multimodal data, separating single modalities and exploring different directions to deepen our understanding of the neuropathology of depression in ADD and MCID.

### Functional imaging abnormalities

Our study observed decreased neural activity in the right SFG in ADD and MCID patients compared with ADND and MCIND patients. The SFG is a core region of the dorsolateral prefrontal cortex and is involved in a variety of functions, such as memory processing, cognitive control, and the reappraisal of negative emotions (Li et al., 2013; McDonald et al., 2020). Our research points to a potential link between the SFG and depression in AD and MCI patients. Moreover, previous studies indicated that cortical thickness in the SFG correlated with irritability symptoms (Cotta Ramusino et al., 2024), potentially reflecting compensatory changes in the SFG during the early stages of the disease. A task fMRI study found that depression is associated with hypoactivation of the SFG during working memory performance (Schiller et al., 2013), suggesting that abnormal activity of the SFG may represent a trait endophenotype for depression. A previous study found that the functional connectivity of the SFG was associated with cognitive deficits in patients with depression (Guan C. et al., 2022), which may contribute to potential neural correlates for cognitive impairment and depressive symptoms. Several previous studies showed that the ADD group showed hypoperfusion in bilateral SFG compared to the ADND group (Akiyama et al., 2008; Hirono et al., 1998), which is consistent with our meta-analysis and provides a framework for understanding the neurophysiological events underlying depressive symptoms in AD.

We found decreased neural activity in the left ITG in the ADD and MCID patients compared with the ADND and MCIND patients. The ITG is involved in face perception and the recognition of objects, and dysfunctional facial emotion processing is one of the clinical features of depression (Lafer-Sousa and Conway, 2013). A previous PET study found a correlation between more severe depressive symptoms and more significant tau accumulation in the ITG (Gatchel et al., 2017), indicating that tau-mediated neurodegeneration in the AD vulnerability region of the ITG may be a potential mechanism for depression. A previous study found that repetitive transcranial magnetic stimulation enhanced bidirectional causal connectivity between the seed and SFG, and between ITG and the seed was significantly improved and correlated with depressive symptoms (Guan M. Z. et al., 2022), suggesting that stronger fronto-temporal coupling may be associated with clinical improvement, and may constitute the neural mechanisms underlying depression. Combined with our findings, these findings may indicate that AD and MCI with depression may lead to an overlapping impairment of neural activity in corresponding brain regions.

Evidence from previous neuroimaging studies has shown that decreased neural activity in the SFG and ITG has been associated with depression in AD and MCI (Yuksel et al., 2018; Ahmad et al., 2023), providing insight into the role of the SFG and ITG in the neural underpinnings of depression. Several rs-fMRI studies have found abnormal functional connectivity between the SFG and brain regions involved in emotional and cognitive processes, including the entorhinal cortex and locus coeruleus in patients with ADD (Dai et al., 2023; Zhu et al., 2022). Moreover, a study investigating the efficacy of donepezil in AD found significantly increased ALFF values in the left SFG after pharmacologic treatment (Zheng et al., 2021), suggesting that the SFG may serve as a potential target for monitoring therapeutic efficacy. Combined with our findings, we suppose that the SFG and ITG are critical regions for depression in ADD and MCID, indicating that depressive symptoms in AD and MCI are owing to a specific pathogenesis but not a psychologically reactive phenomenon.

### Structural imaging abnormalities

We found decreased GMV in the right hippocampus in ADD and MCID compared to ADND and MCIND. The hippocampus is central in memory functions, making it a crucial brain region that mediates both dysregulated mood and cognitive dysfunction (Sampath et al., 2017), contributing to cognitive dysfunction in ADD and MCID. Emerging evidence suggests that atrophy of the hippocampus may be involved in the potential mechanisms underlying the associations among depression, MCI, and AD (Chi et al., 2024). A previous study found that both AD and depression are linked to reduced bilateral hippocampal volume, with AD showing greater atrophy in the left anterior hippocampus, contributing to neural underpinnings of AD and depression (Boccia et al., 2015), which is consistent with our findings. Moreover, evidence indicated that neuroimaging and postmortem studies have consistently identified pathological changes in the hippocampus as being linked to depression in AD patients (Zhang et al., 2024), suggesting hippocampal dysfunction represents a potential neuropathological feature linking depression and cognitive impairment. The findings suggest that atrophy of the hippocampus in ADD and MCID may be associated with the neuropathology of brain dysfunction underlying depression in ADD and MCID.

### Subgroup analysis findings

Our subgroup analysis revealed decreased neural activity in the left SFG in the ADD group compared to the ADND group and in the right SFG and left ITG in the MCID patients compared with the MCIND patients. A previous study showed significantly increased ALFF in the SFG after transcutaneous auricular vagus nerve stimulation in depression patients, which indicated that the SFG may represent a potential neural substrate associated with depressive symptoms (Sun et al., 2022). In our meta-analysis, one study focused on subcortical vascular MCI (Chen et al., 2025), underscoring the importance of addressing mood disturbances in patients with vascular brain injury. Moreover, several fMRI studies found that MCID patients showed decreased functional connectivity between the left ITG and hippocampus compared to MCIND patients (Xu et al., 2021). Moreover, several PET studies found that the glucose metabolism of SFG and ITG was lower in depression patients and correlated with the severity of depression (Fu et al., 2017; Grambal et al., 2009), suggesting that SFG and ITG are involved in neuropathological mechanisms of emotion regulation. These findings highlight that ADD and MCID may have distinct patterns of neural activity, contributing to further understanding of the neural mechanisms underlying depression in ADD and MCID.

### Limitations

There are several limitations in the current meta-analysis. First, the number of included studies and the sample sizes are relatively small, especially for MCI and its subgroups, which may affect the robustness and generalizability of the results, and increase the risks of false positives. Moreover, we strictly applied inclusion criteria, potential publication bias may still affect the analysis results. Second, studies included in this meta-analysis differed in their inclusion criteria of depression in MCI and AD, which is a confounding factor that cannot be overlooked. A more precise and valid definition is needed for future studies, which recruit more homogenous populations and aim better to characterize the behavioral and neural features of depression in AD and MCI. Third, reports on medication use are incomplete in the studies included in this meta-analysis. Medications may directly affect both brain structural and functional alterations, potentially limiting the generalizability of our findings. Fourth, coordinate-based meta-analysis assumed that each voxel in the brain had an equal chance of being activated, potentially overemphasizing the importance of specific brain regions (Tahmasian et al., 2019). There is also a lack of effect-size information and potential reporting bias due to the selective publication of significant coordinates. Fifth, in meta-analysis for fMRI, different imaging modalities (BOLD or SPECT modality) and analysis methods might reflect different facets of neural activation abnormality. Finally, demographic characteristics such as age, disease duration, and disease severity may serve as potential confounding factors. Accordingly, testing different subgroups according to a standardized definition would yield more clinically meaningful findings.

## Conclusion

In conclusion, the multimodal coordinate-based meta-analysis demonstrates abnormalities in structure and neural activity in the bilateral SFG, left ITG, and right HIP in ADD and MCID compared to ADND and MCIND. The findings suggest that neural activity of fronto-temporal regions in patients with ADD and MCID is distinctive from AD and MCI, which may not only shed light on the neuropathology of brain dysfunction underlying depression in AD and MCI but also may provide a framework for future studies to investigate the specific roles of these brain regions in emotion dysregulation.

## Footnotes

1
https://www.brainmap.org/ale/

2
rii.uthscsa.edu/mango/
