# Supplementary material for: Structural and functional brain alterations in depression with Alzheimer’s disease and mild cognitive impairment: a multimodal coordinate-based meta-analysis
Source: Front Aging Neurosci. 2026 Mar 26;18:1784966. doi: 10.3389/fnagi.2026.1784966 (PMC13062311; doi:10.3389/fnagi.2026.1784966)
Supplement: Supplementary file 1 [file Data_Sheet_1.DOCX]

**Supplementary Materials**

**Structural and functional brain alterations in depression with Alzheimer's disease and mild cognitive impairment: A multimodal coordinate-based meta-analysis**

**Supplementary Table 1. Quality assessment checklist.**

| Category 1: Subjects |
| --- |
| 1. Patients were evaluated prospectively, specific diagnostic criteria were applied, and demographic data was reported |
| 2. Healthy comparison subjects were evaluated prospectively, psychiatric and medical illnesses were excluded, and demographic data was reported |
| 3. Important variables (e.g. sample size, age, sex, years of education, and mini-mental state examination (MMSE)) were checked either by stratification or statistically |
| 4. Sample size per group > 10, and no significant difference in age and sex existed |
| Category 2: Methods for image acquisition and analysis |
| 5. Magnet strength at least 1.5T |
| 6. Whole brain analysis was automated with no a-priori regional selection |
| 7. Coordinates reported in a standard space |
| 8. The imaging technique used was clearly described so as it could be reproduced |
| 9. Measurements were clearly described so that they could be reproduced |
| 10. Results have been corrected for multiple comparison |
| Category 3: Results and conclusions |
| 11. Statistical parameters for significant and important non-significant differences were provided |
| 12. Conclusions were consistent with the results obtained and the limitations were discussed |

**Supplementary Table 2. 12-points checklist for included studies**

| 12-points checklist |  | Category 1 | | | |  | Category 2 | | | | | |  | Category 3 | |  | Total scores |
| --- | --- | --- | --- | --- | --- | --- | --- | --- | --- | --- | --- | --- | --- | --- | --- | --- | --- |
|  |  | 1 | 2 | 3 | 4 |  | 5 | 6 | 7 | 8 | 9 | 10 |  | 11 | 12 |  |  |
| (Liao et al., 2003) |  | 1 | 1 | 1 | 0.5 |  | 1 | 1 | 1 | 1 | 1 | 1 |  | 1 | 1 |  | 11.5 |
| (Holthoff et al., 2005) |  | 1 | 1 | 0.5 | 1 |  | 1 | 1 | 1 | 1 | 1 | 0.5 |  | 1 | 1 |  | 11 |
| (Lee et al., 2006) |  | 1 | 1 | 1 | 0.5 |  | 1 | 1 | 1 | 1 | 1 | 1 |  | 1 | 1 |  | 11.5 |
| (Levy et al., 2008) |  | 1 | 1 | 1 | 1 |  | 1 | 1 | 1 | 1 | 1 | 1 |  | 1 | 1 |  | 12 |
| (Lee et al., 2010) |  | 1 | 1 | 1 | 1 |  | 1 | 1 | 1 | 1 | 0.5 | 0.5 |  | 1 | 1 |  | 11 |
| (Xie et al., 2012) |  | 1 | 1 | 1 | 1 |  | 1 | 1 | 1 | 1 | 0.5 | 0.5 |  | 1 | 1 |  | 11 |
| (Kang et al., 2012) |  | 1 | 1 | 1 | 0.5 |  | 1 | 1 | 1 | 1 | 1 | 1 |  | 1 | 1 |  | 11.5 |
| (Son et al., 2013) |  | 1 | 1 | 1 | 1 |  | 1 | 1 | 1 | 1 | 1 | 0.5 |  | 1 | 1 |  | 11.5 |
| (Lebedeva et al., 2014) |  | 1 | 1 | 1 | 1 |  | 1 | 1 | 1 | 1 | 0.5 | 1 |  | 1 | 1 |  | 11.5 |
| (Honda et al., 2014) |  | 1 | 1 | 0.5 | 1 |  | 1 | 1 | 1 | 1 | 1 | 0.5 |  | 1 | 1 |  | 11 |
| (Terada, 2014) |  | 1 | 1 | 1 | 1 |  | 1 | 1 | 1 | 1 | 1 | 0.5 |  | 1 | 1 |  | 11.5 |
| (Oshima et al., 2014) |  | 1 | 1 | 1 | 1 |  | 1 | 1 | 1 | 1 | 1 | 1 |  | 1 | 1 |  | 12 |
| (Brendel et al., 2015) |  | 1 | 1 | 0.5 | 1 |  | 1 | 1 | 1 | 1 | 1 | 0.5 |  | 1 | 1 |  | 11 |
| (Hu et al., 2015) |  | 1 | 1 | 1 | 1 |  | 1 | 1 | 1 | 1 | 1 | 0.5 |  | 1 | 1 |  | 11.5 |
| (Guo et al., 2015) |  | 1 | 1 | 1 | 1 |  | 1 | 1 | 1 | 1 | 1 | 1 |  | 1 | 1 |  | 12 |
| (Hayashi et al., 2016) |  | 1 | 1 | 1 | 1 |  | 1 | 1 | 1 | 1 | 0.5 | 1 |  | 1 | 1 |  | 11.5 |
| (Guo et al., 2017) |  | 1 | 1 | 1 | 1 |  | 1 | 1 | 1 | 1 | 1 | 1 |  | 1 | 1 |  | 12 |
| (Karavasilis et al., 2017) |  | 1 | 1 | 1 | 1 |  | 1 | 1 | 1 | 1 | 1 | 1 |  | 1 | 1 |  | 12 |
| (Liu et al., 2017) |  | 1 | 1 | 1 | 1 |  | 1 | 1 | 1 | 1 | 1 | 1 |  | 1 | 1 |  | 12 |
| (Li et al., 2017) |  | 1 | 1 | 1 | 1 |  | 1 | 1 | 1 | 1 | 1 | 0.5 |  | 1 | 1 |  | 11.5 |
| (Liu et al., 2018) |  | 1 | 1 | 1 | 1 |  | 1 | 1 | 1 | 1 | 1 | 1 |  | 1 | 1 |  | 12 |
| (Lyu et al., 2019) |  | 1 | 1 | 1 | 1 |  | 1 | 1 | 1 | 1 | 1 | 1 |  | 1 | 1 |  | 12 |
| (Yu et al., 2019) |  | 1 | 1 | 1 | 1 |  | 1 | 1 | 1 | 1 | 1 | 1 |  | 1 | 1 |  | 12 |
| (Liu et al., 2019) |  | 1 | 1 | 1 | 1 |  | 1 | 1 | 1 | 1 | 1 | 1 |  | 1 | 1 |  | 12 |
| (Mu et al., 2020) |  | 1 | 1 | 1 | 1 |  | 1 | 1 | 1 | 1 | 1 | 1 |  | 1 | 1 |  | 12 |
| (Mohamed et al., 2021) |  | 1 | 1 | 1 | 1 |  | 1 | 1 | 1 | 1 | 1 | 0.5 |  | 1 | 1 |  | 11.5 |
| (Du et al., 2022) |  | 1 | 1 | 1 | 1 |  | 1 | 1 | 1 | 1 | 1 | 1 |  | 1 | 1 |  | 12 |
| (Hirao et al., 2022) |  | 1 | 1 | 1 | 1 |  | 1 | 1 | 1 | 1 | 1 | 0.5 |  | 1 | 1 |  | 11.5 |
| (Querry et al., 2024) |  | 1 | 1 | 0.5 | 1 |  | 1 | 1 | 1 | 1 | 1 | 0.5 |  | 1 | 1 |  | 11 |
| (Chen et al., 2025) |  | 1 | 1 | 1 | 1 |  | 1 | 1 | 1 | 1 | 1 | 1 |  | 1 | 1 |  | 12 |

**Supplementary Table 3. Search strategy for Pubmed**

| Search number | Query | Results |
| --- | --- | --- |
| 18 | (("Depression"[Mesh] OR "Depressive Symptoms"[Title/Abstract] OR "Depressive Symptom"[Title/Abstract]) AND ("Alzheimer Disease"[Mesh] OR "Alzheimer*"[Title/Abstract] OR "AD"[Title/Abstract] OR "Alzheimer's Disease"[Title/Abstract] OR "Alzheimers Disease"[Title/Abstract] OR "Cognitive Dysfunction"[Mesh] OR "Mild Cognitive Impairment"[Title/Abstract] OR "MCI"[Title/Abstract] OR "Mild Cognitive Impairments"[Title/Abstract] OR "Mild Neurocognitive Disorder"[Title/Abstract] OR "Prodromal Alzheimer*"[Title/Abstract]) AND ("Neuroimaging"[Mesh] OR "Brain Imaging"[Title/Abstract] OR "Brain Image"[Title/Abstract] OR "Magnetic Resonance Imaging"[Mesh] OR "MR Imaging"[Title/Abstract] OR "Magnetic Resonance Image"[Title/Abstract] OR "MRI Scans"[Title/Abstract] OR "MRI Scan"[Title/Abstract] OR "Positron Emission Tomography"[Title/Abstract] OR "PET"[Title/Abstract] OR "Single-Photon Emission Computed Tomography"[Title/Abstract] OR "SPECT"[Title/Abstract] OR "fMRI"[Title/Abstract] OR "Functional MRI"[Title/Abstract] OR "Functional Magnetic Resonance Imaging"[Title/Abstract] OR "sMRI"[Title/Abstract] OR "structural MRI"[Title/Abstract] OR "structural Magnetic Resonance Imaging"[Title/Abstract])) AND ("Humans"[Mesh]) AND ("1995"[Date - Publication] : "2025"[Date - Publication]) | 555 |
| 17 | (("Depression"[Mesh] OR "Depressive Symptoms"[Title/Abstract] OR "Depressive Symptom"[Title/Abstract]) AND ("Alzheimer Disease"[Mesh] OR "Alzheimer*"[Title/Abstract] OR "AD"[Title/Abstract] OR "Alzheimer's Disease"[Title/Abstract] OR "Alzheimers Disease"[Title/Abstract] OR "Cognitive Dysfunction"[Mesh] OR "Mild Cognitive Impairment"[Title/Abstract] OR "MCI"[Title/Abstract] OR "Mild Cognitive Impairments"[Title/Abstract] OR "Mild Neurocognitive Disorder"[Title/Abstract] OR "Prodromal Alzheimer*"[Title/Abstract]) AND ("Neuroimaging"[Mesh] OR "Brain Imaging"[Title/Abstract] OR "Brain Image"[Title/Abstract] OR "Magnetic Resonance Imaging"[Mesh] OR "MR Imaging"[Title/Abstract] OR "Magnetic Resonance Image"[Title/Abstract] OR "MRI Scans"[Title/Abstract] OR "MRI Scan"[Title/Abstract] OR "Positron Emission Tomography"[Title/Abstract] OR "PET"[Title/Abstract] OR "Single-Photon Emission Computed Tomography"[Title/Abstract] OR "SPECT"[Title/Abstract] OR "fMRI"[Title/Abstract] OR "Functional MRI"[Title/Abstract] OR "Functional Magnetic Resonance Imaging"[Title/Abstract] OR "sMRI"[Title/Abstract] OR "structural MRI"[Title/Abstract] OR "structural Magnetic Resonance Imaging"[Title/Abstract])) AND ("Humans"[Mesh]) | 562 |
| 16 | (("Depression"[Mesh] OR "Depressive Symptoms"[Title/Abstract] OR "Depressive Symptom"[Title/Abstract]) AND ("Alzheimer Disease"[Mesh] OR "Alzheimer*"[Title/Abstract] OR "AD"[Title/Abstract] OR "Alzheimer's Disease"[Title/Abstract] OR "Alzheimers Disease"[Title/Abstract] OR "Cognitive Dysfunction"[Mesh] OR "Mild Cognitive Impairment"[Title/Abstract] OR "MCI"[Title/Abstract] OR "Mild Cognitive Impairments"[Title/Abstract] OR "Mild Neurocognitive Disorder"[Title/Abstract] OR "Prodromal Alzheimer*"[Title/Abstract]) AND ("Neuroimaging"[Mesh] OR "Brain Imaging"[Title/Abstract] OR "Brain Image"[Title/Abstract] OR "Magnetic Resonance Imaging"[Mesh] OR "MR Imaging"[Title/Abstract] OR "Magnetic Resonance Image"[Title/Abstract] OR "MRI Scans"[Title/Abstract] OR "MRI Scan"[Title/Abstract] OR "Positron Emission Tomography"[Title/Abstract] OR "PET"[Title/Abstract] OR "Single-Photon Emission Computed Tomography"[Title/Abstract] OR "SPECT"[Title/Abstract] OR "fMRI"[Title/Abstract] OR "Functional MRI"[Title/Abstract] OR "Functional Magnetic Resonance Imaging"[Title/Abstract] OR "sMRI"[Title/Abstract] OR "structural MRI"[Title/Abstract] OR "structural Magnetic Resonance Imaging"[Title/Abstract])) | 601 |
| 15 | ("Neuroimaging"[Mesh] OR "Brain Imaging"[Title/Abstract] OR "Brain Image"[Title/Abstract] OR "Magnetic Resonance Imaging"[Mesh] OR "MR Imaging"[Title/Abstract] OR "Magnetic Resonance Image"[Title/Abstract] OR "MRI Scans"[Title/Abstract] OR "MRI Scan"[Title/Abstract] OR "Positron Emission Tomography"[Title/Abstract] OR "PET"[Title/Abstract] OR "Single-Photon Emission Computed Tomography"[Title/Abstract] OR "SPECT"[Title/Abstract] OR "fMRI"[Title/Abstract] OR "Functional MRI"[Title/Abstract] OR "Functional Magnetic Resonance Imaging"[Title/Abstract] OR "sMRI"[Title/Abstract] OR "structural MRI"[Title/Abstract] OR "structural Magnetic Resonance Imaging"[Title/Abstract]) | 945,777 |
| 14 | "MR Imaging"[Title/Abstract] OR "Magnetic Resonance Image"[Title/Abstract] OR "Magnetic Resonance Imaging"[Title/Abstract] OR "MRI Scans"[Title/Abstract] OR "MRI Scan"[Title/Abstract] OR "Positron Emission Tomography"[Title/Abstract] OR "PET"[Title/Abstract] OR "Single-Photon Emission Computed Tomography"[Title/Abstract] OR "SPECT"[Title/Abstract] OR "fMRI"[Title/Abstract] OR "Functional MRI"[Title/Abstract] OR "Functional Magnetic Resonance Imaging"[Title/Abstract] OR "sMRI"[Title/Abstract] OR "structural MRI"[Title/Abstract] OR "structural Magnetic Resonance Imaging"[Title/Abstract] | 639,530 |
| 13 | "Magnetic Resonance Imaging"[Mesh] | 591,638 |
| 12 | "Neuroimaging"[Mesh] OR "Brain Imaging"[Title/Abstract] OR "Brain Image"[Title/Abstract] | 229,106 |
| 11 | "Brain Imaging"[Title/Abstract] OR "Brain Image"[Title/Abstract] | 22,197 |
| 10 | "Neuroimaging"[Mesh] | 212,107 |
| 9 | ("Cognitive Dysfunction"[Mesh] OR "Mild Cognitive Impairment"[Title/Abstract] OR "MCI"[Title/Abstract] OR "Mild Cognitive Impairment"[Title/Abstract] OR "Mild Cognitive Impairments"[Title/Abstract] OR "Mild Neurocognitive Disorder"[Title/Abstract] OR "Prodromal Alzheimer*"[Title/Abstract]) | 78,919 |
| 8 | "Mild Cognitive Impairment"[Title/Abstract] OR "MCI"[Title/Abstract] OR "Mild Cognitive Impairment"[Title/Abstract] OR "Mild Cognitive Impairments"[Title/Abstract] OR "Mild Neurocognitive Disorder"[Title/Abstract] OR "Prodromal Alzheimer*"[Title/Abstract] | 42,918 |
| 7 | "Cognitive Dysfunction"[Mesh] | 53,211 |
| 6 | ("Alzheimer Disease"[Mesh] OR "Alzheimer*"[Title/Abstract] OR "AD"[Title/Abstract] OR "Alzheimer's Disease"[Title/Abstract] OR "Alzheimers Disease"[Title/Abstract]) | 349,938 |
| 5 | "Alzheimer*"[Title/Abstract] OR "AD"[Title/Abstract] OR "Alzheimer's Disease"[Title/Abstract] OR "Alzheimers Disease"[Title/Abstract] | 337,629 |
| 4 | "Alzheimer Disease"[Mesh] | 139,390 |
| 3 | ("Depression"[Mesh] OR "Depressive Symptoms"[Title/Abstract] OR "Depressive Symptom"[Title/Abstract]) | 222,818 |
| 2 | "Depressive Symptoms"[Title/Abstract] OR "Depressive Symptom"[Title/Abstract] | 84,605 |
| 1 | "Depression"[Mesh] | 178,733 |

**Supplementary Table 4. The Medication treatment of the included articles**

| Study | Anti-dementia drug | Antidepressant | Note | | |
| --- | --- | --- | --- | --- | --- |
| (Lebedeva et al., 2014) | Not reported | Not reported |  | | |
| (Hu et al., 2015) | Not reported | Not reported |  | | |
| (Karavasilis et al., 2017) | Not reported | No |  | | |
| (Mohamed et al., 2021) | Not reported | Not reported |  | | |
| (Querry et al., 2024) | Not reported | Yes | 29.7% of AD patients were taking an antidepressant | | |
| (Xie et al., 2012) | ChEI/MEM | Yes | 14 MCI (ChEI: N = 9; MEM: N = 4; combined: N = 1),  5 MCID (SSRI: N = 2, SNRI: N = 2, and bupropion: N = 1) | | |
| (Lyu et al., 2019) | Not reported | Not reported |  | | |
| (Du et al., 2022) | Not reported | Not reported |  | | |
| (Chen et al., 2025) | Not reported | Not reported |  | | |
| (Holthoff et al., 2005) | ChEI | No | 19 AD patients were taking ChEI and one received psychotropic medication | | |
| (Lee et al., 2006) | donepezil | No | two (one ADD vs. one ADND) were taking donepezil, stopped the medication for 2 weeks before imaging | | |
| (Levy et al., 2008) | ChEI | Yes | 18 AD patients (11 ADD vs. 7 ADND) were taking ChEI,  9 (5 ADD vs. 4 ADND) were taking antidepressants | | |
| (Kang et al., 2012) | Not reported | Not reported |  | | |
| (Oshima et al., 2014) | ChEI | No | 57 AD (23 ADD vs. 34 ADND) were taking ChEI | | |
| (Terada et al., 2014) | No | No |  | | |
| (Honda et al., 2014) | No | No |  | | |
| (Guo et al., 2015) | Not reported | Not reported |  | | |
| (Hayashi et al., 2016) | No | No |  | | |
| (Guo et al., 2017) | Not reported | Not reported |  | | |
| (Liu et al., 2017) | Not reported | Not reported |  | | |
| (Mu et al., 2020) | Not reported | Not reported |  | | |
| (Lee et al., 2010) | Not reported | Not reported |  | | |
| (Brendel et al., 2015) | Not reported | Yes | 62 % of MCID and 5 % of MCIND were taking SSRI | | |
| (Li et al., 2017) | Not reported | Not reported |  | | |
| (Liu et al., 2018) | Not reported | No |  | | |
| (Yu et al., 2019) | Not reported | Not reported |  | | |
| (Liu et al., 2019) | Not reported | Not reported |  | | |
| (Hirao et al., 2022) | No | No |  | | |
|  |  |  | |  |  |

**Supplementary Table 5. Jackknife analyses for brain areas showing alterations in ADD and MCID compared to ADND and MCIND.**

| Jackknife analyses discarded study  (first author, year) | Brain regions (MNI coordinates) | | | |
| --- | --- | --- | --- | --- |
|  | R superior frontal gyrus (32,50,26) | L superior frontal gyrus (-22,8,58) | L inferior temporal gyrus (-54,-54,-2) | R hippocampus (30,-8,-20) |
| (Liao et al., 2003) | Yes | Yes | Yes | Yes |
| (Holthoff et al., 2005) | Yes | Yes | Yes | No |
| (Lee et al., 2006) | Yes | Yes | Yes | Yes |
| (Levy et al., 2008) | Yes | Yes | Yes | Yes |
| (Lee et al., 2010) | Yes | Yes | Yes | No |
| (Xie et al., 2012) | Yes | Yes | No | Yes |
| (Kang et al., 2012) | Yes | Yes | Yes | Yes |
| (Son et al., 2013) | Yes | Yes | Yes | Yes |
| (Lebedeva et al., 2014) | Yes | Yes | Yes | Yes |
| (Honda et al., 2014) | No | Yes | Yes | Yes |
| (Terada, 2014) | Yes | Yes | Yes | Yes |
| (Oshima et al., 2014) | Yes | Yes | Yes | Yes |
| (Brendel et al., 2015) | Yes | No | Yes | No |
| (Hu et al., 2015) | Yes | Yes | Yes | Yes |
| (Guo et al., 2015) | Yes | No | Yes | Yes |
| (Hayashi et al., 2016) | Yes | Yes | Yes | Yes |
| (Guo et al., 2017) | Yes | Yes | Yes | Yes |
| (Karavasilis et al., 2017) | Yes | Yes | Yes | Yes |
| (Liu et al., 2017) | Yes | Yes | Yes | No |
| (Li et al., 2017) | Yes | Yes | Yes | Yes |
| (Liu et al., 2018) | Yes | Yes | Yes | Yes |
| (Lyu et al., 2019) | Yes | Yes | Yes | Yes |
| (Yu et al., 2019) | Yes | Yes | Yes | Yes |
| (Liu et al., 2019) | Yes | Yes | No | Yes |
| (Mu et al., 2020) | Yes | Yes | Yes | Yes |
| (Mohamed et al., 2021) | Yes | Yes | Yes | Yes |
| (Du et al., 2022) | Yes | Yes | Yes | Yes |
| (Hirao et al., 2022) | Yes | Yes | Yes | Yes |
| (Querry et al., 2024) | Yes | Yes | No | Yes |
| (Chen et al., 2025) | Yes | Yes | Yes | Yes |
|  | 29/30 | 28/30 | 27/30 | 26/30 |

**Supplementary Table 6. Jackknife analyses for modality meta-analyses in functional imaging studies of ADD and MCID**

| Jackknife analyses discarded study  (first author, year) | Brain regions (MNI coordinates) | |
| --- | --- | --- |
|  | R superior frontal gyrus L inferior temporal gyrus  (32,50,26) (-54,-54,-2) | |
| (Holthoff et al., 2005) | Yes | Yes |
| (Lee et al., 2006) | No | Yes |
| (Levy et al., 2008) | Yes | No |
| (Lee et al., 2010) | Yes | Yes |
| (Kang et al., 2012) | Yes | Yes |
| (Son et al., 2013) | Yes | Yes |
| (Honda et al., 2014) | Yes | Yes |
| (Terada, 2014) | No | Yes |
| (Oshima et al., 2014) | Yes | Yes |
| (Brendel et al., 2015) | Yes | Yes |
| (Guo et al., 2015) | Yes | No |
| (Hayashi et al., 2016) | Yes | No |
| (Guo et al., 2017) | Yes | Yes |
| (Liu et al., 2017) | Yes | Yes |
| (Li et al., 2017) | Yes | Yes |
| (Liu et al., 2018) | Yes | Yes |
| (Yu et al., 2019) | Yes | Yes |
| (Liu et al., 2019) | Yes | Yes |
| (Mu et al., 2020) | Yes | Yes |
| (Hirao et al., 2022) | Yes | Yes |
|  | 18/20 | 17/20 |

**Supplementary Table 7. Jackknife analyses for structural imaging modality meta-analyses in ADD and MCID**

| Jackknife analyses Brain regions (MNI coordinates) discarded study R hippocampus  (first author, year) (32,50,26) | |
| --- | --- |
|  | |
| (Xie et al., 2012) | Yes |
| (Son et al., 2013) | Yes |
| (Lebedeva et al., 2014) | No |
| (Hu et al., 2015) | Yes |
| (Karavasilis et al., 2017) | Yes |
| (Lyu et al., 2019) | Yes |
| (Mohamed et al., 2021) | Yes |
| (Du et al., 2022) | No |
| (Querry et al., 2024) | Yes |
| (Chen et al., 2025) | Yes |
|  | 8/10 |

**Supplementary Table 8. Jackknife analyses for subgroup meta-analyses of ADD compared to ADND.**

| Jackknife analyses Brain regions (MNI coordinates) discarded study R hippocampus  (first author, year) (32,50,26) | |
| --- | --- |
|  | |
| (Son et al., 2013) | Yes |
| (Lebedeva et al., 2014) | Yes |
| (Hu et al., 2015) | No |
| (Karavasilis et al., 2017) | Yes |
| (Mohamed et al., 2021) | Yes |
| (Querry et al., 2024) | Yes |
| (Liao et al., 2003) | No |
| (Holthoff et al., 2005) | Yes |
| (Lee et al., 2006) | Yes |
| (Levy et al., 2008) | Yes |
| (Kang et al., 2012) | Yes |
| (Honda et al., 2014) | Yes |
| (Terada, 2014) | Yes |
| (Oshima et al., 2014) | Yes |
| (Guo et al., 2015) | Yes |
| (Hayashi et al., 2016) | Yes |
| (Guo et al., 2017) | Yes |
| (Liu et al., 2017) | Yes |
| (Mu et al., 2020) | Yes |
|  | 17/19 |
|  |  |

**Supplementary Table 9. Jackknife analyses for subgroup meta-analyses in MCID compared to MCIND.**

| Jackknife analyses discarded study  (first author, year) | Brain regions (MNI coordinates) | |
| --- | --- | --- |
|  | R superior frontal gyrus L inferior temporal gyrus  (32,50,26) (-54,-54,-2) | |
| (Lee et al., 2010) | Yes | Yes |
| (Xie et al., 2012) | No | Yes |
| (Brendel et al., 2015) | Yes | No |
| (Li et al., 2017) | Yes | Yes |
| (Liu et al., 2018) | Yes | Yes |
| (Lyu et al., 2019) | Yes | Yes |
| (Liu et al., 2019) | Yes | No |
| (Yu et al., 2019) | Yes | Yes |
| (Hirao et al., 2022) | Yes | Yes |
| (Du et al., 2022) | Yes | Yes |
| (Chen et al., 2025) | Yes | Yes |
|  | 10/11 | 9/11 |

| Analysis | Cluster | side | Brain region | BA | Coordinates (Talairach)  X Y Z | | | Cluster size (mm^3^) | ALE  value |
| --- | --- | --- | --- | --- | --- | --- | --- | --- | --- |
| ADND > ADD | 1 | Left | SFG | 9 | 32 | 50 | 26 | 388 | 0.0158 |
| MCIND>MCID | 1 | Right | SFG | 6 | -22 | 8 | 58 | 504 | 0.0203 |
|  | 2 | Left | ITG | 37 | -54 | -54 | -2 | 560 | 0.0202 |

**Supplementary Table 10. Subgroup meta-analyses in MCID compared to MCIND for individuals older than 65 years.**

**Supplementary Table 11. Subgroup meta-analyses of studies that were not diagnosed with depression using DSM or other**

| Analysis | Cluster | side | Brain region | BA | Coordinates (Talairach)  X Y Z | | | Cluster size (mm^3^) | ALE  value |
| --- | --- | --- | --- | --- | --- | --- | --- | --- | --- |
| **All-effects** | 1 | Right | SFG | 9 | 32 | 50 | 26 | 396 | 0.0203 |
|  | 2 | Left | SFG | 6 | -22 | 8 | 58 | 315 | 0.0148 |
|  | 3 | Left | ITG | 37 | -54 | -54 | -2 | 392 | 0.0202 |
|  | 4 | Right | HIP |  | 30 | -8 | -18 | 272 | 0.0138 |
| **Subgroup** |  |  |  |  |  |  |  |  |  |
| ADND > ADD |  |  |  |  |  |  |  |  |  |
|  | 1 | Left | SFG | 6 | -22 | 8 | 58 | 476 | 0.0148 |
| ADND < ADD | No sig. |  |  |  |  |  |  |  |  |
| **Modality** |  |  |  |  |  |  |  |  |  |
| Functional imaging |  |  |  |  |  |  |  |  |  |
|  | 1 | Right | SFG | 9 | 32 | 50 | 26 | 535 | 0.0203 |
|  | 2 | Left | ITG | 37 | -54 | -54 | -2 | 521 | 0.0202 |
| structural imaging |  |  |  |  |  |  |  |  |  |
|  | 1 | Right | HIP |  | 30 | -8 | -20 | 252 | 0.0118 |
