# Supplementary material for: Structural and functional brain alterations in depression with Alzheimer’s disease and mild cognitive impairment: a multimodal coordinate-based meta-analysis
Source: Front Aging Neurosci. 2026 Mar 26;18:1784966. doi: 10.3389/fnagi.2026.1784966 (PMC13062311; doi:10.3389/fnagi.2026.1784966)

**Supplementary Material 2 Detailed Literature Deduplication**

**Structural and functional brain alterations in depression with Alzheimer's disease and mild cognitive impairment: A multimodal coordinate-based meta-analysis**

**1. Software Used:**

We employed EndNote for the majority of the duplicate identification and removal process. EndNote is a reference management software commonly used in systematic reviews and meta-analyses for managing citations and references.

**2. Duplicate Identification and Removal Process:**

We followed a two-stage approach for duplicate removal: an automatic deduplication process using EndNote’s built-in features, followed by a manual review to ensure accuracy.

**Stage 1: Automatic Deduplication in EndNote**

**Step 1.1: Importing References**

All retrieved articles (555 articles) were imported into EndNote for citation management. This included both records from database searches and articles identified through other means.

**Step 1.2: Initial Duplicate Removal Using EndNote**

We used EndNote’s automatic duplicate detection feature, which identifies duplicate references based on key fields including author, year, and title. During the initial screening, EndNote identified and removed **185 duplicate references**. To ensure transparency and reproducibility, we have included screenshots of the duplicates identified during the automatic removal process.


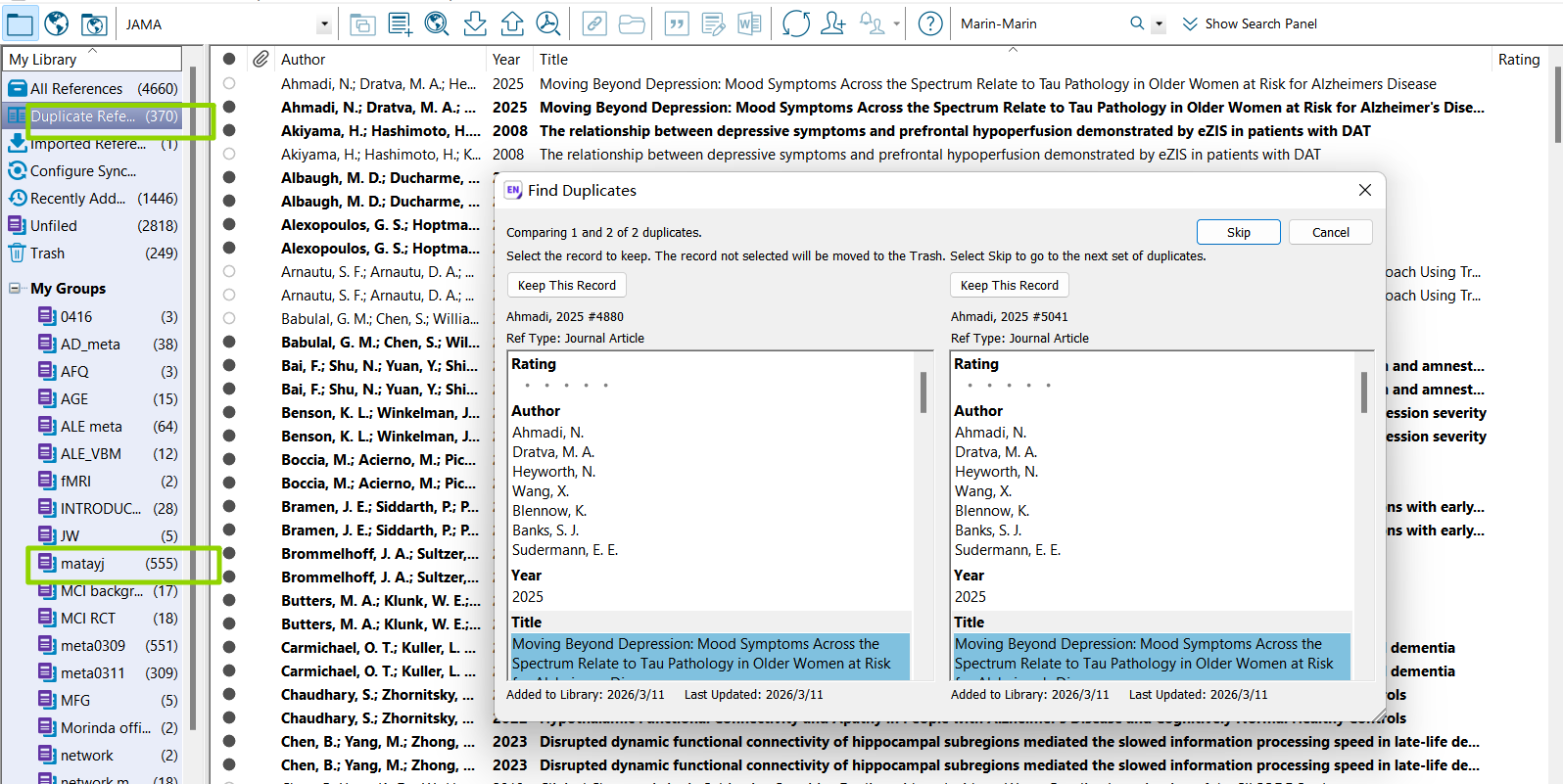


We have included screenshots of the duplicates identified during the automatic removal process. These screenshots clearly show the 185 duplicate references that were flagged and removed.


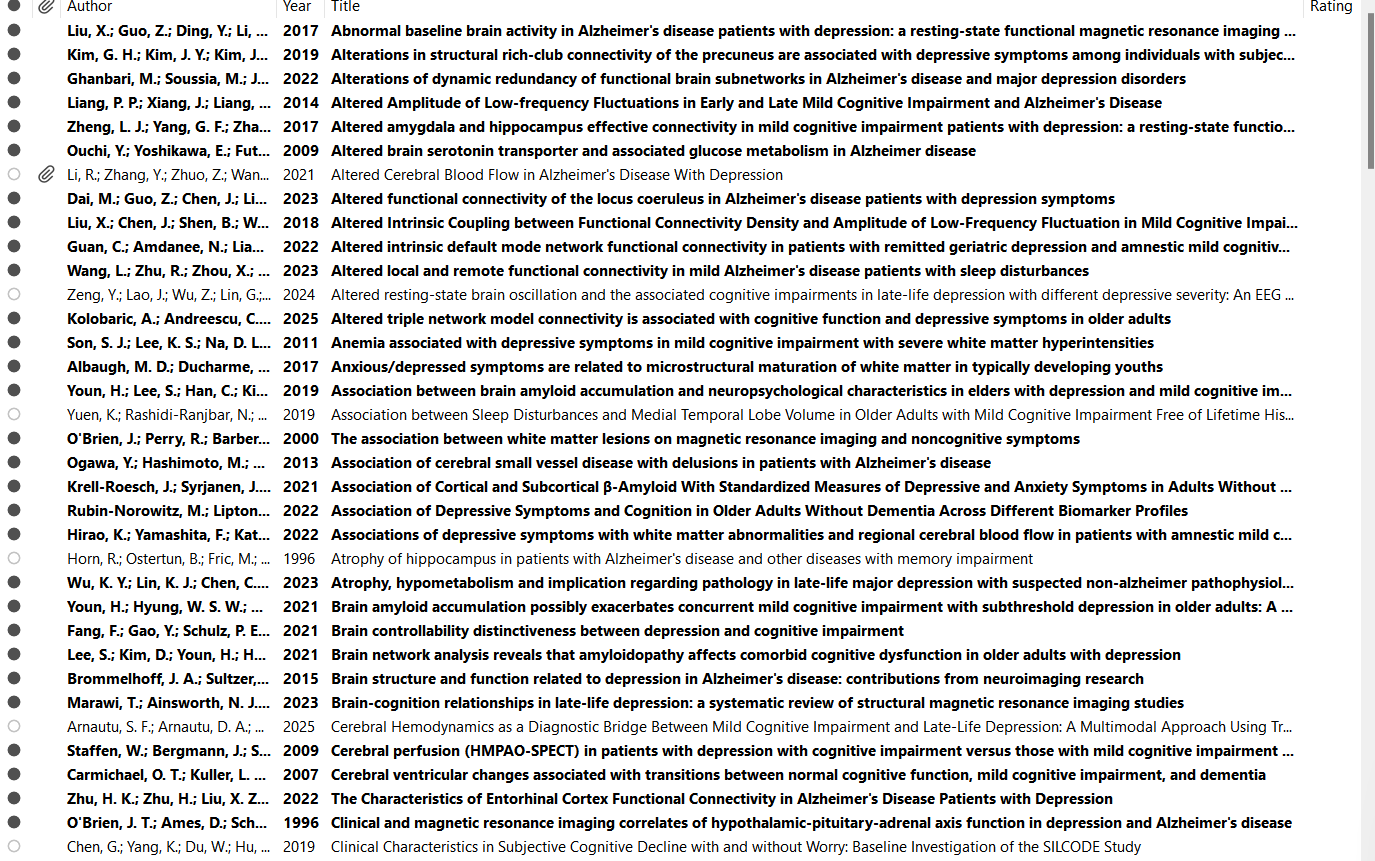

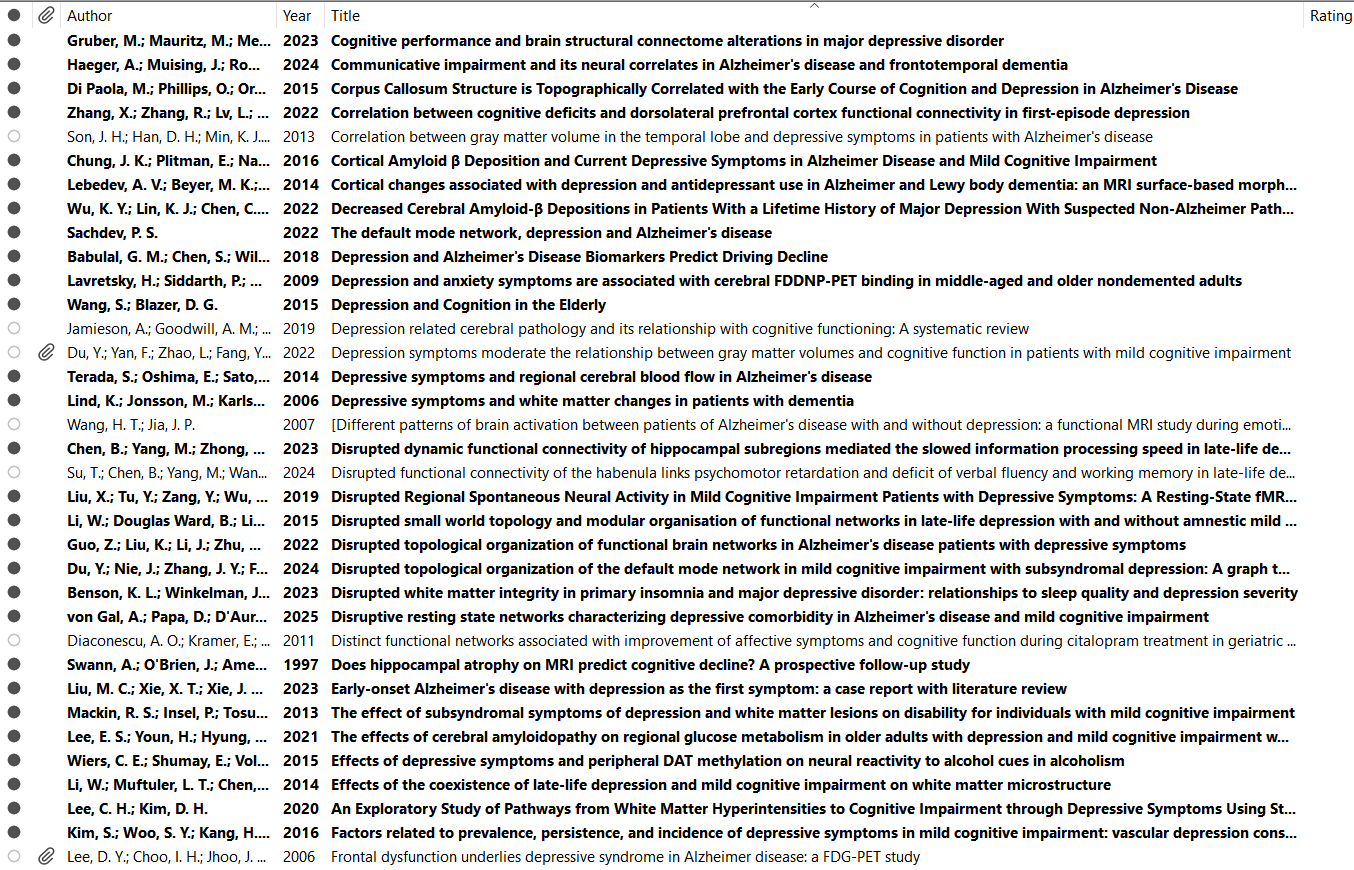


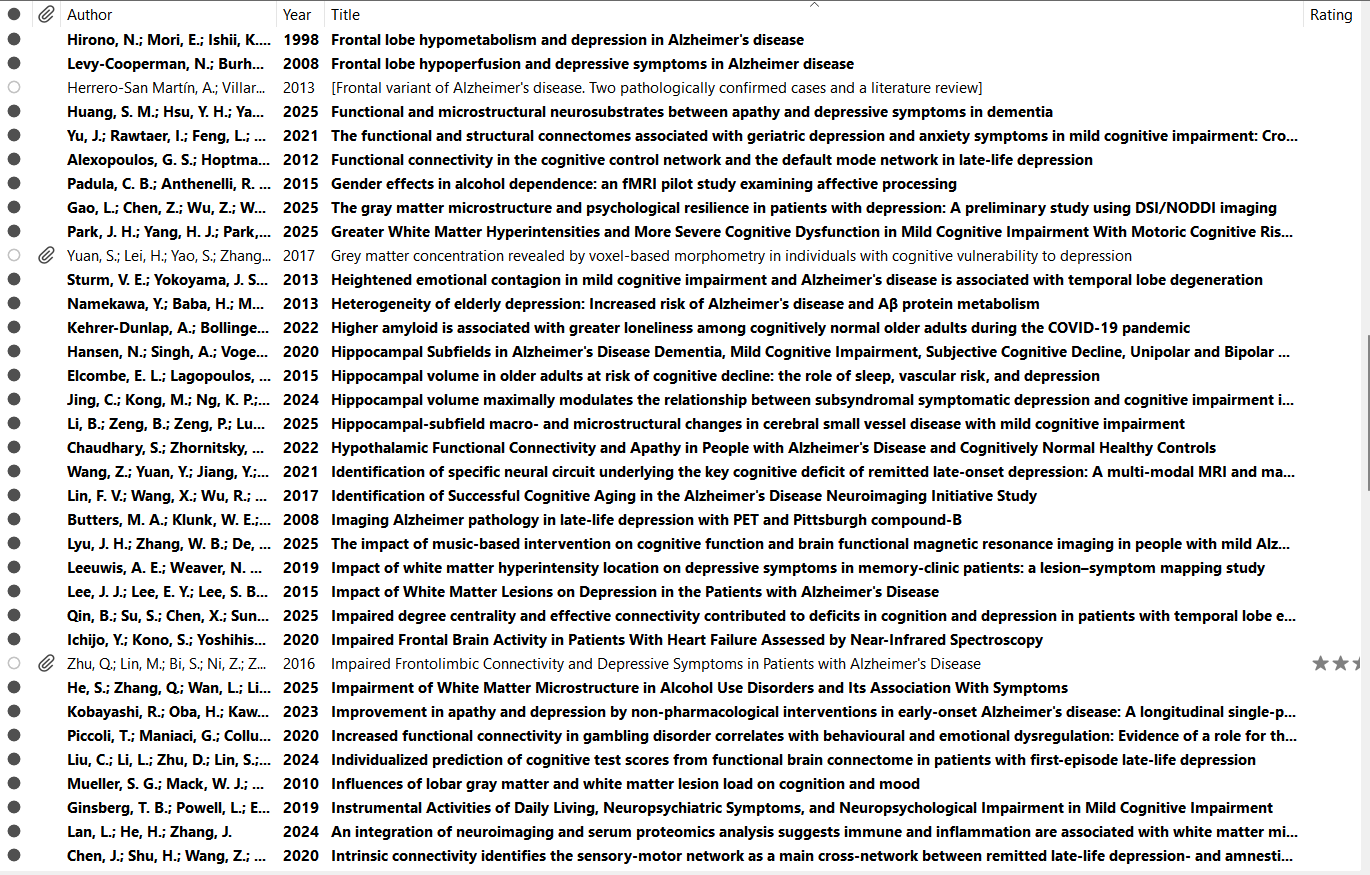

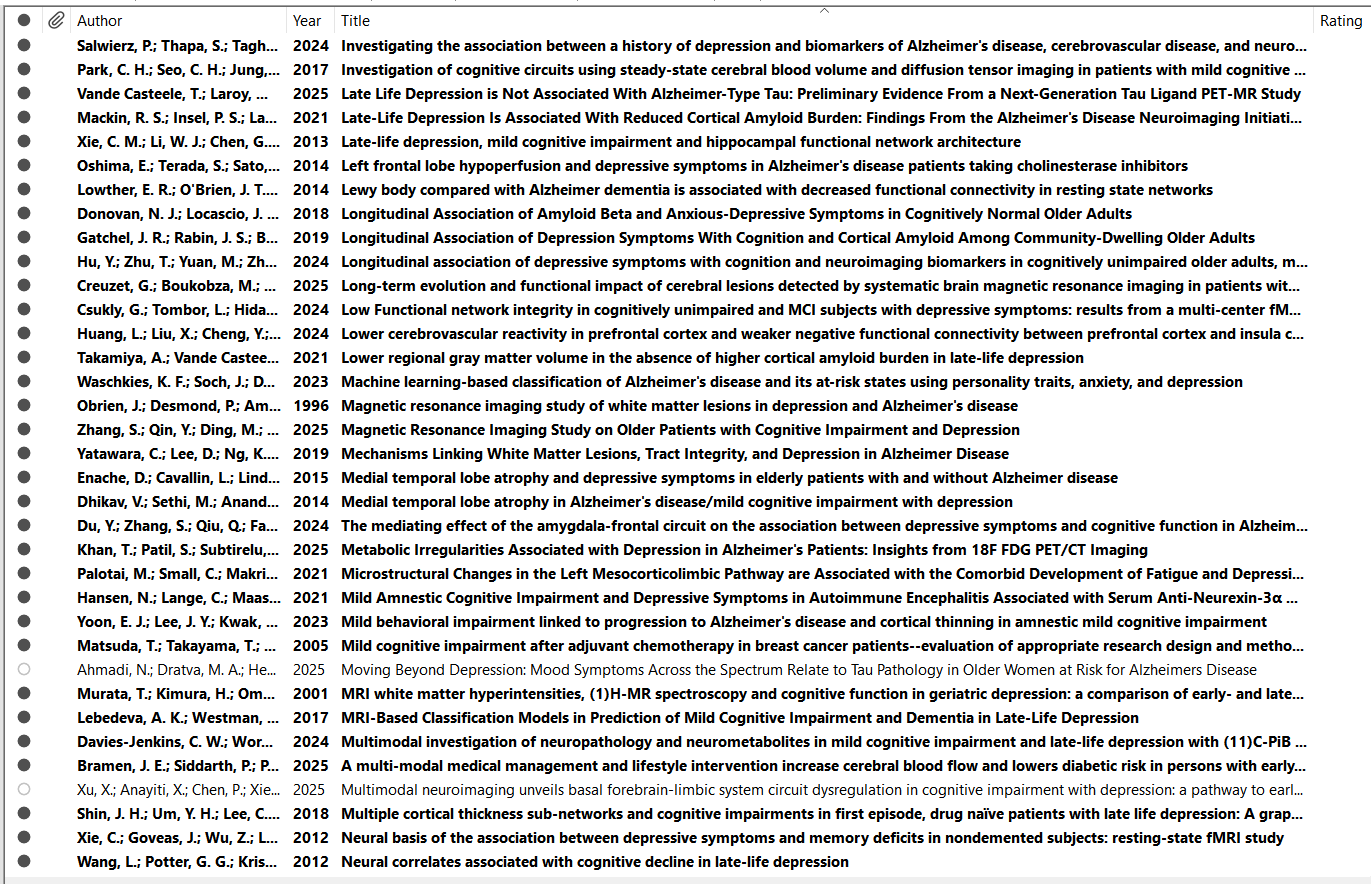


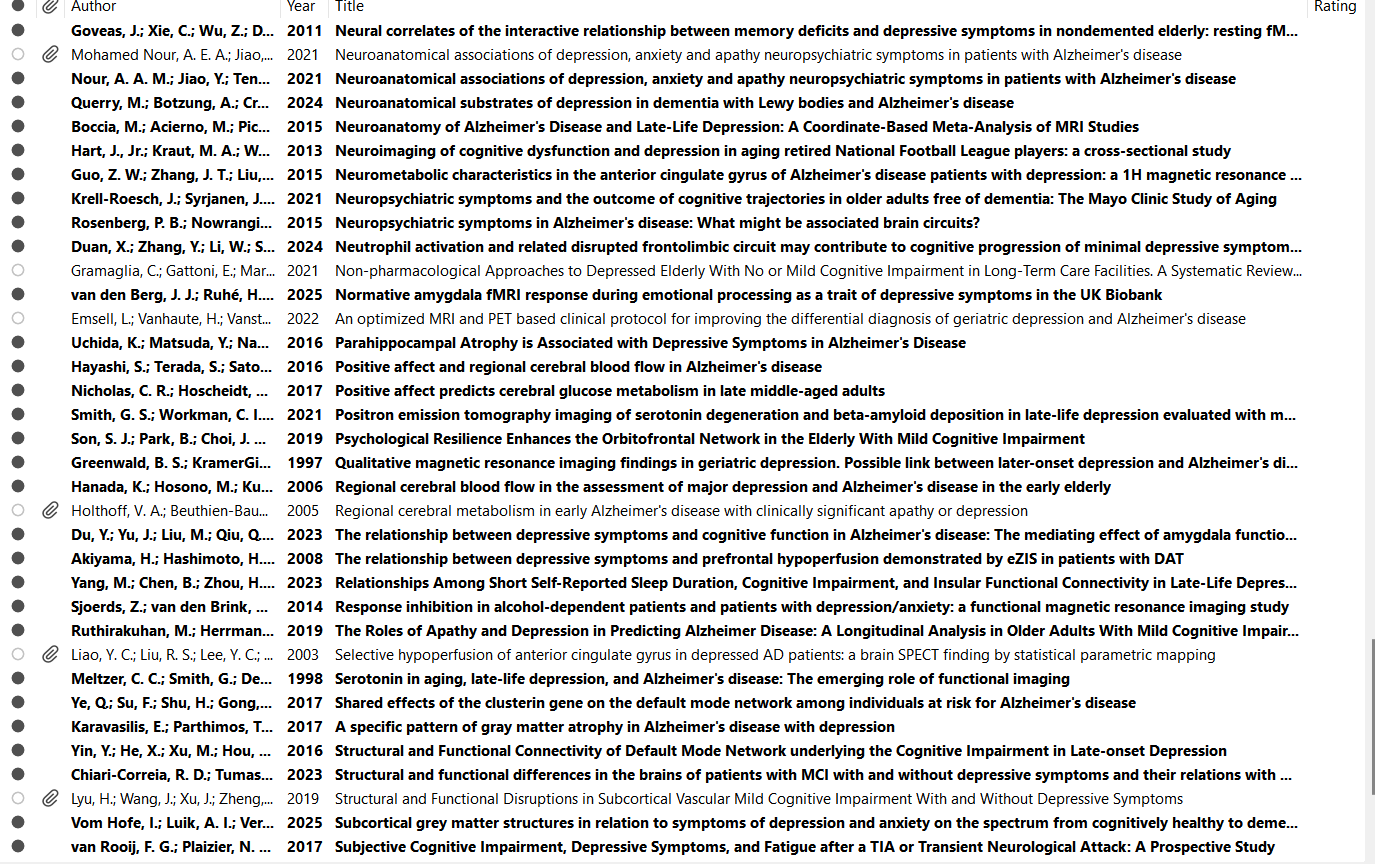


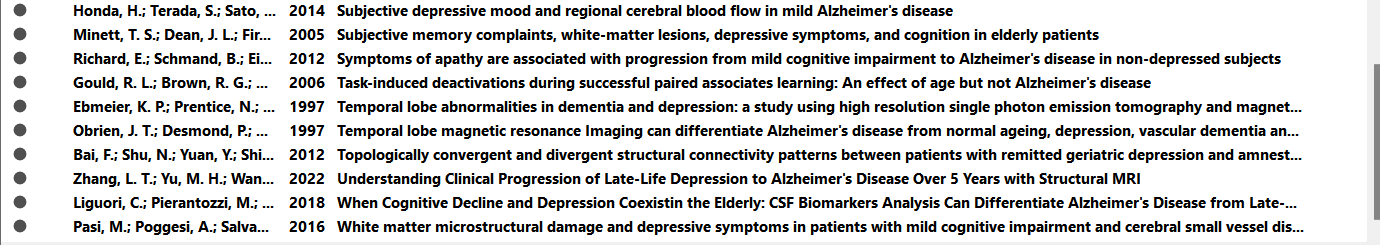


**Stage 2: Manual Review**

**Step 2.1: Review of EndNote’s Automatic Deduplication Results**

After the automatic removal, we carefully reviewed the remaining references. We cross-checked citations that EndNote had flagged as duplicates to ensure accuracy. This step was crucial, as some references may not have been automatically identified due to variations in formatting, incoplete metadata, or slight differences in author names (e.g., initial versus full first names).

**Step 2.2: Manual Screening**

We manually reviewed the remaining **370 references** to identify any potential duplicates that may have been overlooked by EndNote’s automatic deduplication process. This involved a detailed examination of titles, authors, and publication years to ensure the uniqueness of each reference. After performing the automatic deduplication process using EndNote, we manually reviewed **51 references** to ensure that no duplicates were overlooked.


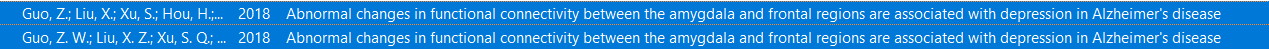


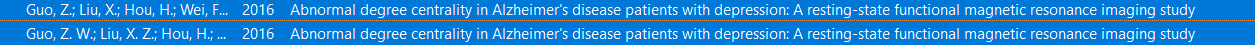


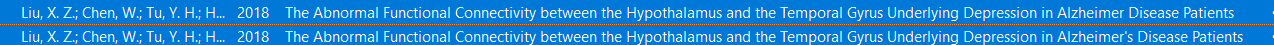


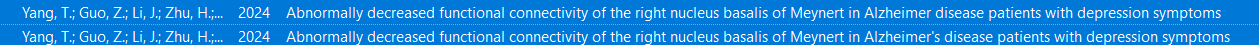


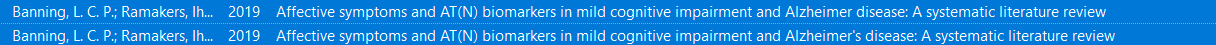


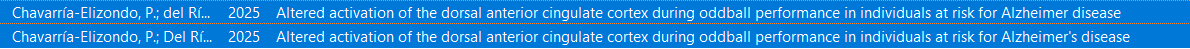


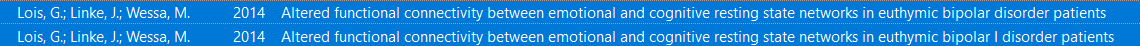


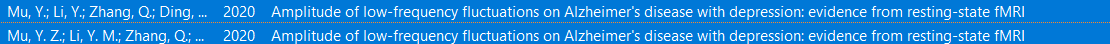


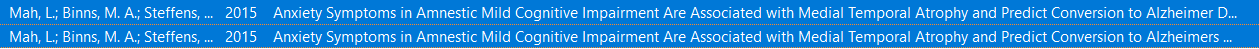


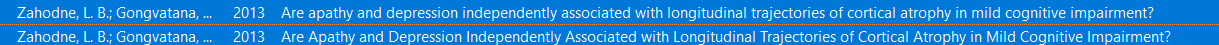


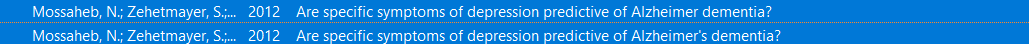


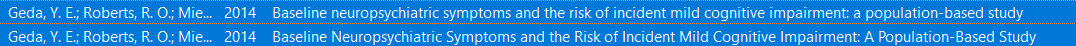


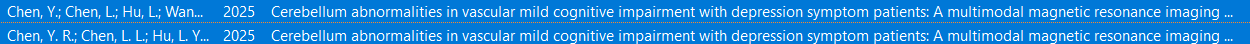


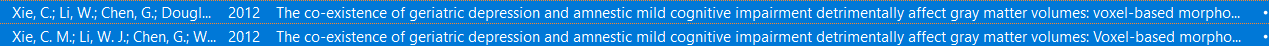


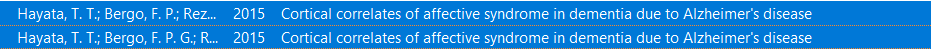


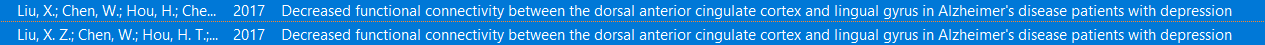


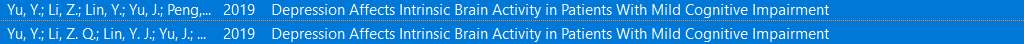


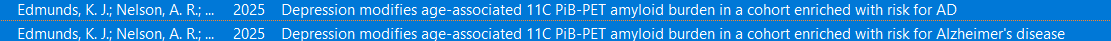


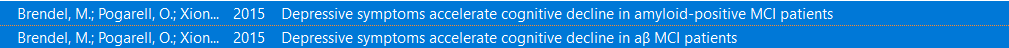


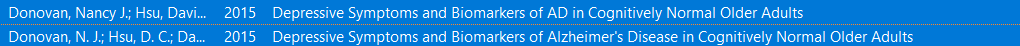


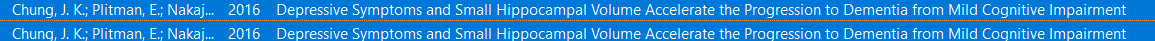


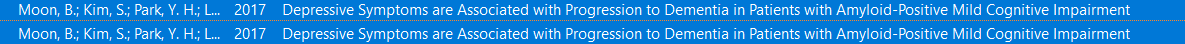


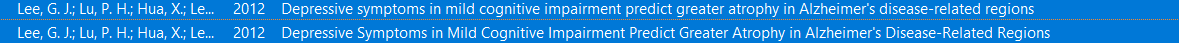


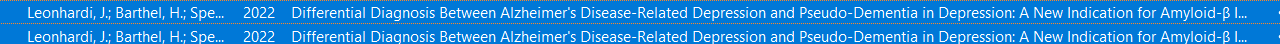


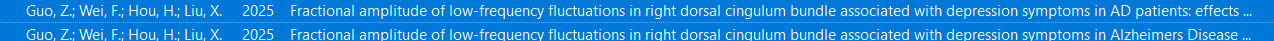


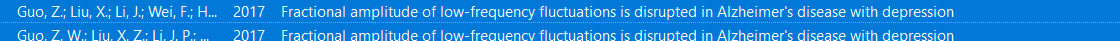


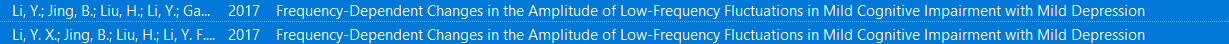


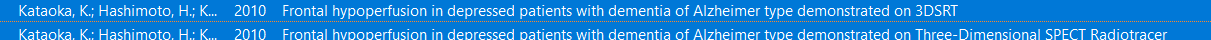


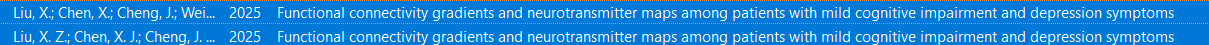


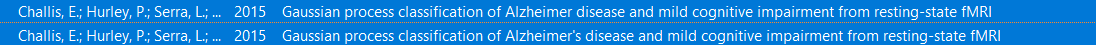


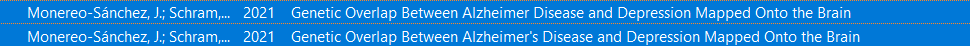


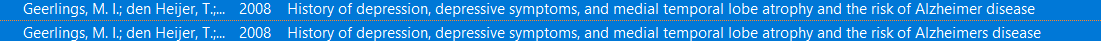


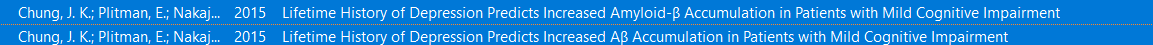


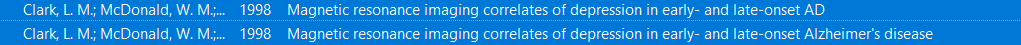


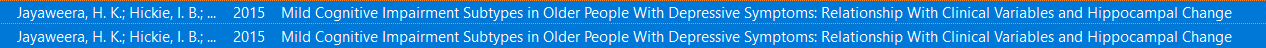


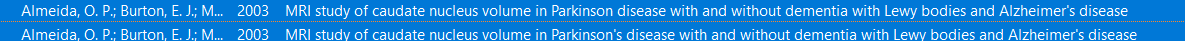


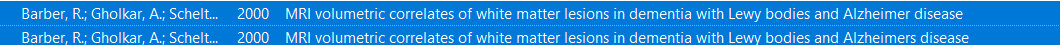


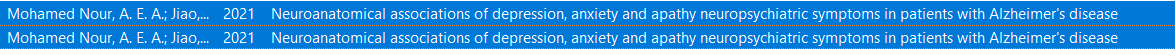


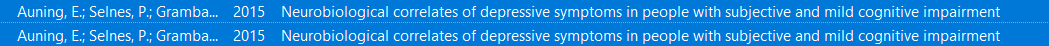


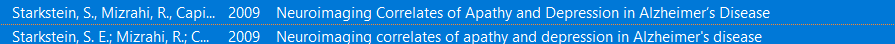


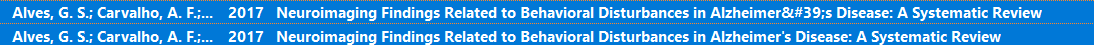


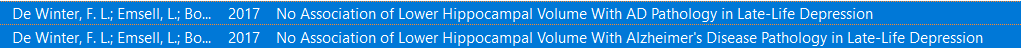


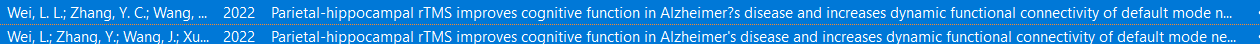


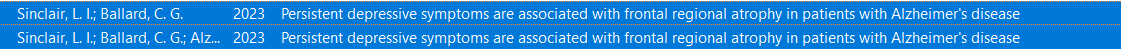


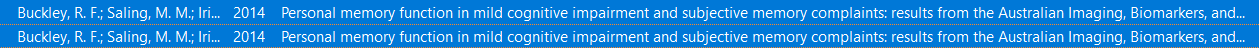


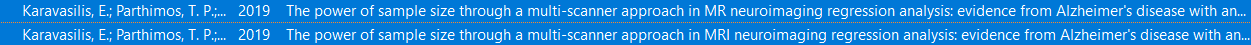


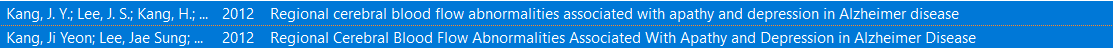


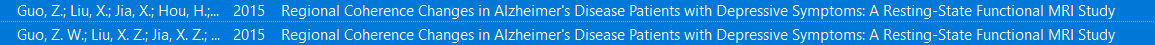


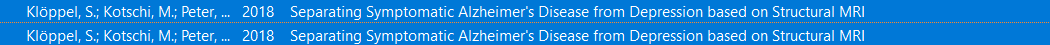


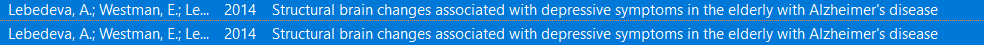


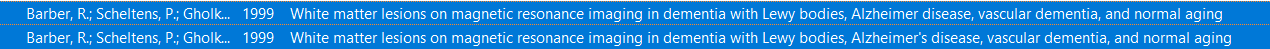


**Step 2.3: Final Removal of Duplicates**

After manually reviewing and cross-checking the remaining **370 references**, we removed **51 duplicates** identified at this stage. The final set of **319 references** was then used for the subsequent stages of the study selection process. We have included screenshots that clearly illustrate the duplicates identified during the deduplication process.


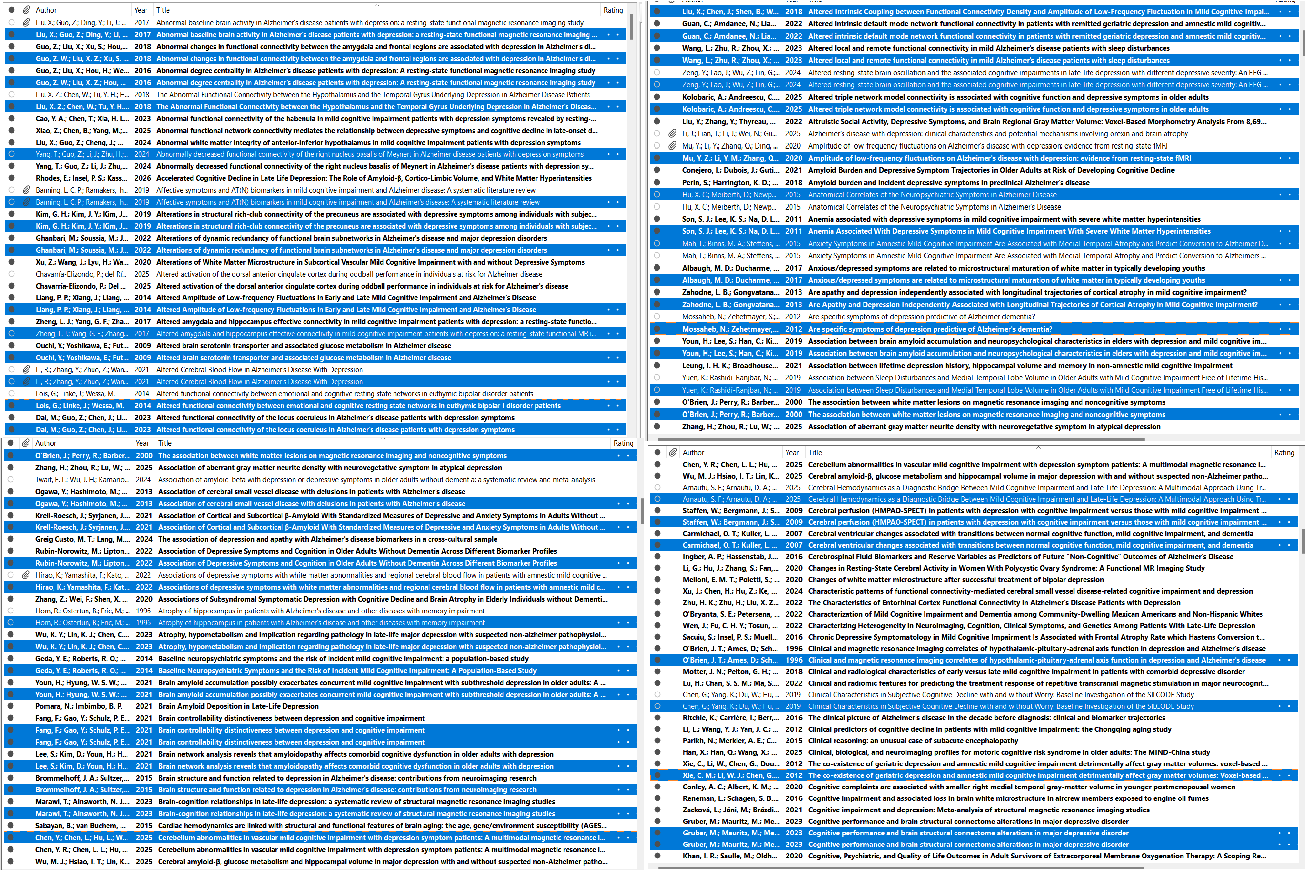


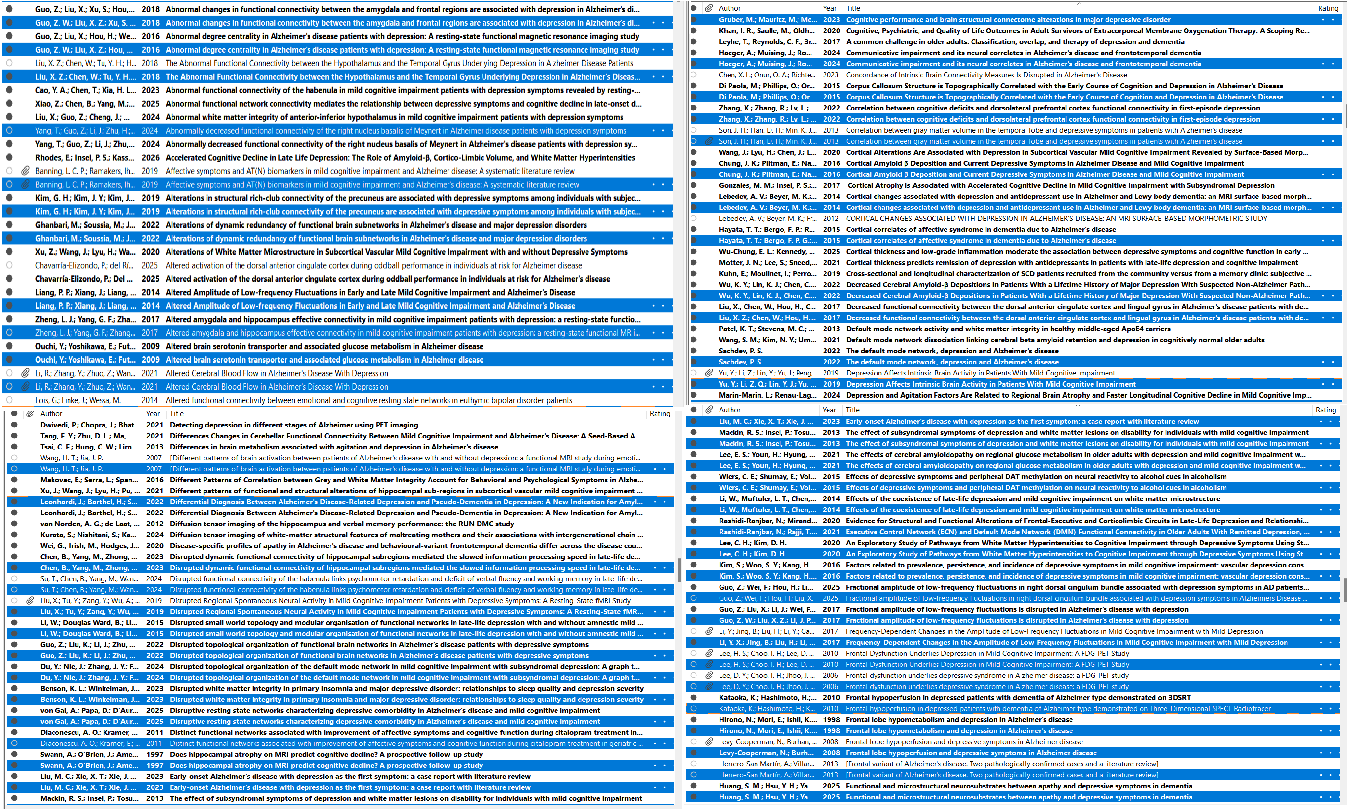


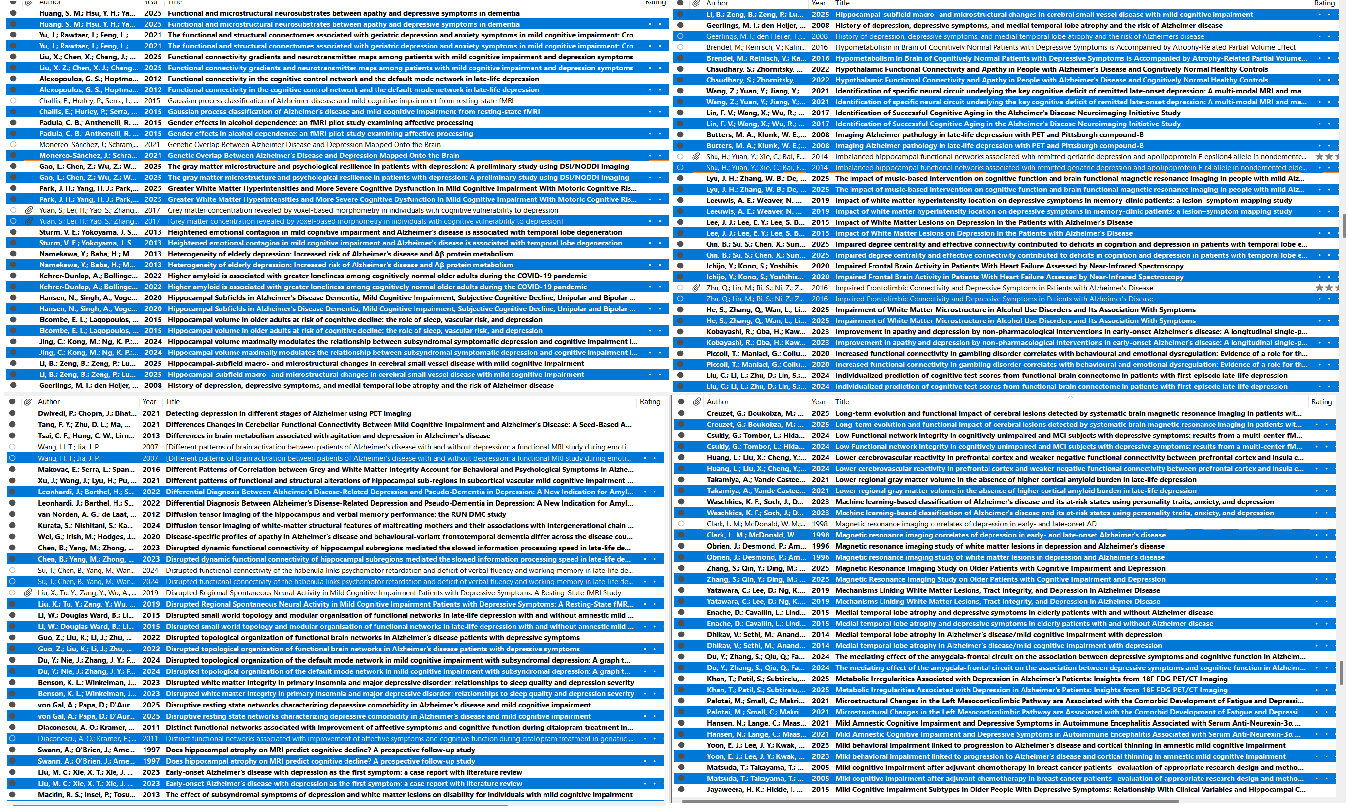


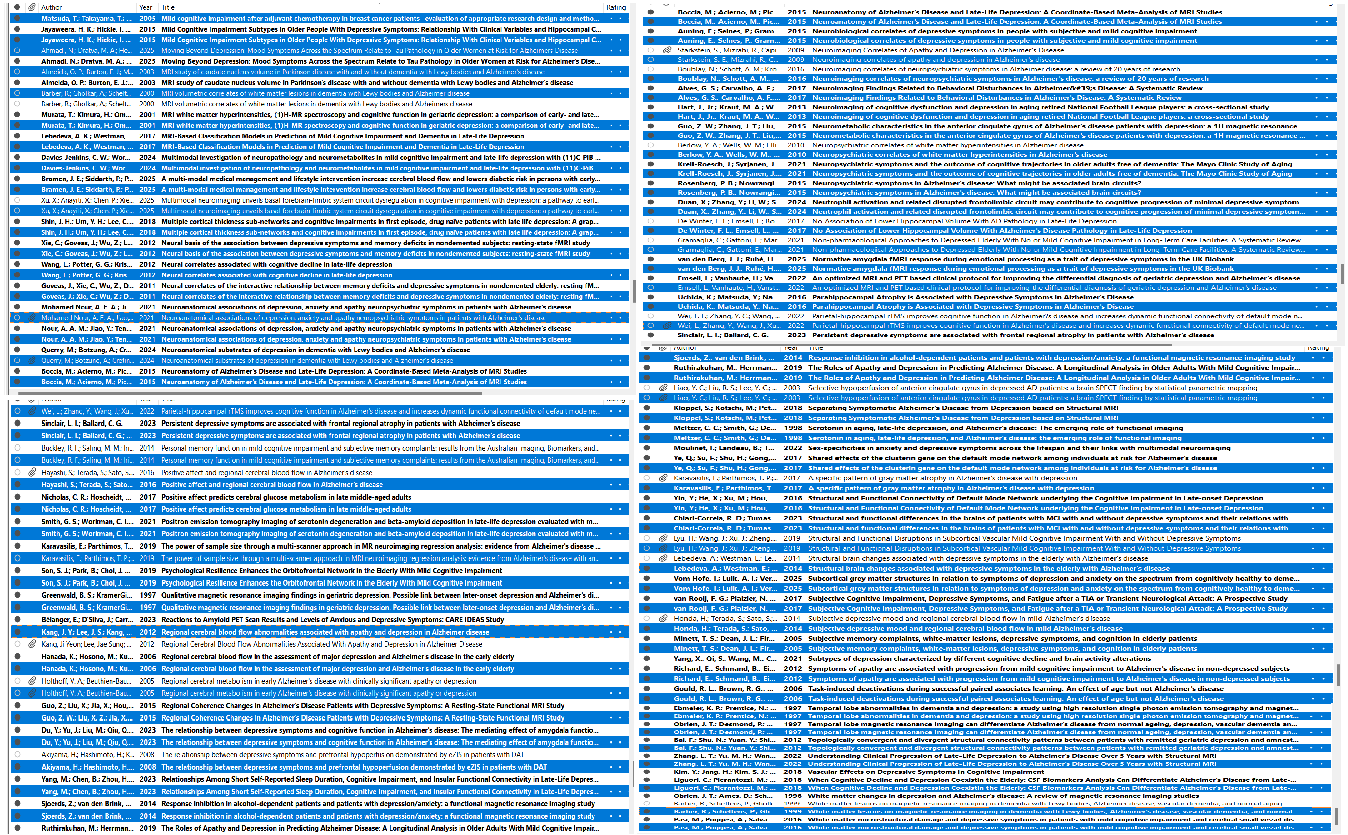

Supplement: Supplementary file 2 [file Data_Sheet_2.DOCX]
